# Multiplatform Metabolomics Reveals Novel Serum Metabolite Biomarkers in Diabetic Retinopathy Subjects

**DOI:** 10.1002/advs.202001714

**Published:** 2020-10-01

**Authors:** Qiuhui Xuan, Yang Ouyang, Yanfeng Wang, Liang Wu, Huating Li, Yuanyuan Luo, Xinjie Zhao, Disheng Feng, Wangshu Qin, Chunxiu Hu, Lina Zhou, Xinyu Liu, Haidong Zou, Chun Cai, Jiarui Wu, Weiping Jia, Guowang Xu

**Affiliations:** ^1^ CAS Key Laboratory of Separation Science for Analytical Chemistry Dalian Institute of Chemical Physics Chinese Academy of Sciences 457 Zhongshan Road Dalian 116023 China; ^2^ University of Chinese Academy of Sciences Beijing 100049 China; ^3^ Shanghai Diabetes Institute, Shanghai Key Laboratory of Diabetes Mellitus, Shanghai Clinical Center for Endocrine and Metabolic Diseases, Metabolic diseases biobank Shanghai JiaoTong University Affiliated Sixth People's Hospital Shanghai 200233 China; ^4^ Department of Ophthalmology First People's Hospital of Shanghai Shanghai Jiao Tong University Shanghai China; ^5^ Key Laboratory of Systems Biology, CAS Center for Excellence in Molecular Cell Science, Institute of Biochemistry and Cell Biology, University of Chinese Academy of Sciences Chinese Academy of Sciences 320 Yue‐Yang Road Shanghai 200031 China; ^6^ Key Laboratory of Systems Biology CAS Center for Excellence in Molecular Cell Science Institute of Biochemistry and Cell Biology University of Chinese Academy of Sciences Chinese Academy of Sciences 320 Yue‐Yang Road Shanghai 200031 China; ^7^ Shanghai Diabetes Institute Shanghai Key Laboratory of Diabetes Mellitus Shanghai Clinical Center for Endocrine and Metabolic Diseases Shanghai Jiaotong University Affiliated Sixth People's Hospital Shanghai 200233 China; ^8^ CAS Key Laboratory of Separation Science for Analytical Chemistry Dalian Institute of Chemical Physics Chinese Academy of Sciences Dalian 116023 China

**Keywords:** diabetic retinopathy, lipidomics, metabolomics, multiplatforms, serum biomarkers

## Abstract

Diabetic retinopathy (DR) is the main cause of vision loss or blindness in working age adults worldwide. The lack of effective diagnostic biomarkers for DR leads to unsatisfactory curative treatments. To define potential metabolite biomarkers for DR diagnosis, a multiplatform‐based metabolomics study is performed. In this study, a total of 905 subjects with diabetes without DR (NDR) and with DR at different clinical stages are recruited. Multiplatform metabolomics methods are used to characterize the serum metabolic profiles and to screen and validate the DR biomarkers. Based on the criteria *p* < 0.05 and false‐discovery rate < 0.05, 348 and 290 metabolites are significantly associated with the pathogenesis of DR and early‐stage DR, respectively. The biomarker panel consisting of 12‐hydroxyeicosatetraenoic acid (12‐HETE) and 2‐piperidone exhibited better diagnostic performance than hemoglobin A1c (HbA1c) in differentiating DR from diabetes, with AUCs of 0.946 versus 0.691 and 0.928 versus 0.648 in the discovery and validation sets, respectively. In addition, this panel showed higher sensitivity in early‐stage DR detection than HbA1c. In conclusion, this multiplatform‐based metabolomics study comprehensively revealed the metabolic dysregulation associated with DR onset and progression. The defined biomarker panel can be used for detection of DR and early‐stage DR.

## Graphical abstract

Multiplatform metabolomics methods are used to comprehensively investigate the association between the serum metabolic profiles and the onset and progression of diabetic retinopathy (DR) in a large Chinese population, and 12‐HETE and 2‐piperidone are identified as a highly effective marker panel for the detection of DR and early‐stage DR.

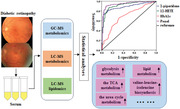

## 1 Introduction

Diabetic retinopathy (DR), as a main microvascular complication of diabetes mellitus (DM), remains a leading cause of vision loss among working aged adults worldwide. The International Diabetes Federation (2015) estimated that the occurrence of DR and vision‐threatening DR would increase to 191.0 and 56.3 million, respectively, by 2030. In addition, the presence of DR indicates an increased risk of life‐threatening systemic vascular complications.^[^ ^1^ ^]^

Screening and early diagnosis of DR are particularly important in the prevention and treatment of this disease. Although retinal imaging methods (such as standard or wide‐field retinal imaging, optical coherence tomography) are commonly utilized to screen and diagnose DR in the clinic and have successfully reduced the rate of vision loss,^[^ ^2^ ^]^ current DR screening is challenged by issues related to availability of primary healthcare workers who are capable of assessing retinal images.^[^ ^3^ ^]^ Therefore, effective method such as reliable biomarkers for screening DR is required to prevent the progression of disease.^[^ ^4^ ^]^ Moreover, retinal vascular and neural damage may occur before evident clinical DR, accompanied by the appearance of microaneurysms and exudates on retinal edema. Hemoglobin A1c (HbA1c) is the only validated systemic biomarker for DR progression;^[^ ^5^ ^]^ good glycemic control could significantly reduce the development of microvascular complications. However, only 6.6% of the variation in the risk of retinopathy was explained by HbA1c.^[^ ^5^ ^]^ Therefore, there is still an urgent need to identify novel biomarkers for DR screening or detection.

Metabolomics is one of the “omics” techniques and is complementary to genomics, transcriptomics, and proteomics. This study aims to provide a method for comprehensive profiling of low‐molecular‐weight metabolites in complex biological matrices. Many studies^[^ ^6^ ^]^ have shown obvious metabolic disorders associated with DM and DM‐related complications. Therefore, metabolomics and lipidomics provide powerful platforms for discovering novel markers and biochemical processes to improve diagnostics, prognosis, and treatment.

Metabolomics studies of DR are still in the early stage. Paris et al. and Wang et al. detected and identified metabolite markers using vitreous samples.^[7^^]^ However, it is not easy to detect DR with vitreous biomarkers due to the invasiveness of vitreous sampling. Some studies^[^ ^8^ ^]^ have also shown serum metabolite biomarkers and dysfunctional pathways associated with DR; nevertheless, further application of these biomarkers in the clinic was restricted by a limited research cohort or lack of validation. Therefore, further study is required to discover and validate biomarkers from large‐scale samples.

Due to the complex genetic and microenvironmental backgrounds of patients, discovering novel biomarkers requires comprehensive metabolomics studies. Nuclear magnetic resonance (NMR) spectroscopy, liquid chromatography‐mass spectrometry (LC‐MS) and gas chromatography‐mass spectrometry(GC‐MS) are the most useful tools for metabolic or lipidomic profiling analysis. NMR spectroscopy is popular because of simple pre‐treatment, non‐destructive analysis, and high reproducibility, but its disadvantages are low detection sensitivity and low coverage of metabolites.^[^ ^9^ ^]^ Chromatography for its superior separation power and MS for its higher universality and sensitivity are playing an increasingly important role in metabolomics. LC‐MS lipidomics (LC‐MSL) focuses on the measurement of lipids and cannot provide data on other metabolites (e.g., amino acids, nucleic acids, bile acids, and saccharides). GC‐MS can be used to analyze volatile and semivolatile metabolites, and reversed‐phase LC‐MS is suitable for hydrophobic metabolites. The combination of multiple platforms, including GC‐MS metabolomics (GC‐MSM), LC‐MS metabolomics (LC‐MSM), and LC‐MSL, is better for detecting as many metabolites as possible.

The goal of this study is to obtain metabolic profiling data from multiple analytical platforms to comprehensively elucidate the abnormal metabolism associated with DR onset and development and to further identify reliable serum biomarkers for the diagnosis of DR and early‐stage DR in the population with diabetes. Thus, a total of 905 participants were enrolled to explore metabolic profiles and abnormal metabolic pathways associated with DR, and a two‐step analysis strategy including discovery and validation studies, was used to discover and validate a novel biomarker panel and test its clinical practicability. Additionally, patients with mild nonproliferative DR (NPDR) were specifically recruited to evaluate the performance of the biomarker panel in differentiating early‐stage DR.

## 2 Results and Discussion

**Figure 1:**
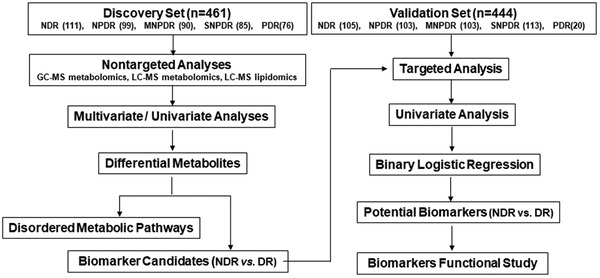
Design of the study.

In this study, using nontargeted GC‐MSM, LC‐MSM, and LC‐MSL platforms, we comprehensively elucidated the metabolic profiles of DR and early‐stage DR and the related disordered metabolism pathways underlying DR development. Subsequently, we identified and validated a novel biomarker panel for differentiating DR and early DR and tested its clinical practicability. A workflow of this study is shown in **Figure** **1**. In the discovery study, all 461 serum samples, including 111 NDR samples and 350 DR samples, were collected to explore abnormal metabolism and dysfunctional pathways of DR compared with NDR and establish reliable biomarker models. Among these DR samples, 99, 90, 85, and 76 cases were diagnosed as NPDR, moderate NPDR (MNPDR), severe NPDR (SNPDR), and proliferative DR (PDR), respectively. In the validation set, a total of 444 serum samples including 105 NDR, 103 NPDR, 103 MNPDR, 113 SNPDR, and 20 PDR samples, were used to test a defined biomarker panel and evaluate its diagnostic performance for DR and early‐stage DR. Last, the influence of 2‐piperidone on human retinal endothelial cells (hRECs) was investigated to explore whether it is related to DR onset and progression.

Nontargeted profiling was performed for the discovery set on the three different platforms (GC‐MSM, LC‐MSM, and LC‐MSL) to obtain the serum metabolic characteristics as comprehensively as possible. The relative standard derivation (RSD) of the distribution for the detected metabolites in the quality control (QC) serum samples inserted into the analysis batches is shown in Figure S1, Supporting Information, and the data show that the present analyses were reliable. Finally, 139 metabolites were identified by a library search (NIST, FiehnLib and our in‐house database) and retention index verification by GC‐MSM; 194 metabolites were identified based on our in‐house database containing more than 2000 metabolite standards^[^ ^10^ ^]^ with LC‐MSM; and 484 lipid species were identified based on our in‐house lipid database, including the retention time (t*_R_*), exact *m*/*z* and/or MS characteristic fragments,^[^ ^11^ ^]^ with LC‐MSL. The identified metabolites included organic acids (such as citrate, isocitrate, succinate, fumarate, and malate), amino acids (e.g., lysine, beta‐alanine, alanine, threonine, aspartic acid, glutamine, glutamic acid, citrulline, ornithine, tryptophan, tyrosine, phenylalanine, valine, and leucine), nucleosides and their derivatives (e.g., hypoxanthine, xanthine, and uridine), saccharides and their derivatives (such as fructose, mannitol, mannose, maltose, arabitol, and lyxose), carnitines, glycerides, sphingolipids, phospholipids, and fatty acids. The subsequent statistical analyses were performed using the metabolites with less than 30% RSD.

### 2.1 Metabolic and Lipid Profiling of DR

**Figure 2:**
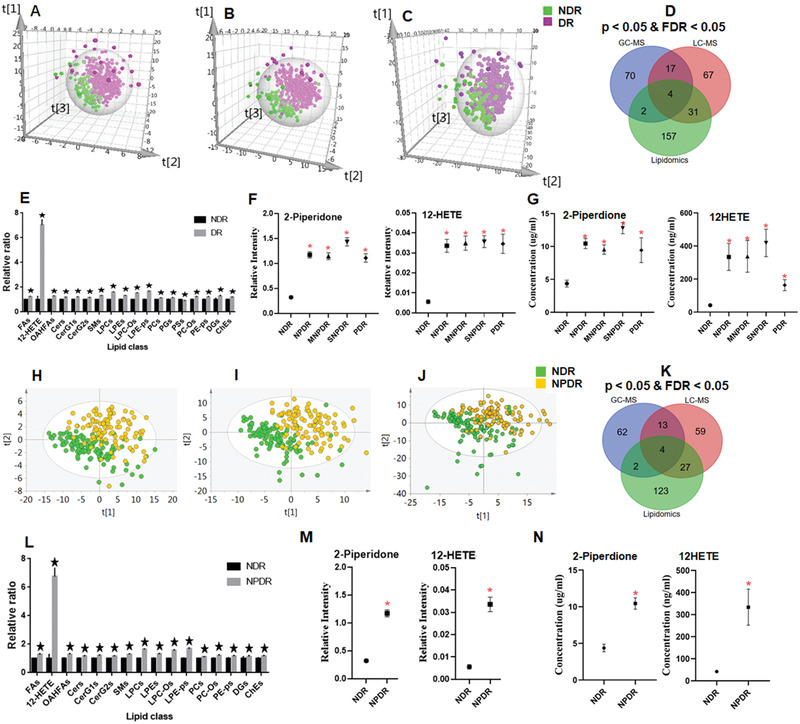
Partial least squares discriminant analysis score plots for NDR and DR, including the NPDR, MNPDR, SNPDR, and PDR groups in the discovery set from A) GC‐MSM, B) LC‐MSM, and C) LC‐MSL. D) Venn diagram displaying the differential metabolites when the DR group was compared with the NDR group in the discovery set based on GC‐MSM, LC‐MSM, and LC‐MSL. E) Serum relative concentrations of significantly differential lipid (sub)classes based on LC‐MSL in the discovery set. F) Serum concentrations of 12‐HETE and 2‐piperidone based on the LC‐MS platform in the discovery and G) validation sets. PLS‐DA score plots for NDR and NPDR groups in the discovery set from H) GC‐MSM, I) LC‐MSM, and J) LC‐MSL. K) Venn diagram displaying the differential metabolites when the NPDR group was compared with the NDR group in the discovery set based on GC‐MSM, LC‐MSM, and LC‐MSL. L) Relative serum concentrations of significantly differential lipid (sub)classes based on LC‐MSL in the discovery set. M) Serum concentrations of 12‐HETE and 2‐piperidone based on the LC‐MS platform in the discovery and N) validation sets. The metabolite data were compared using nonparametric tests in Wilcoxon, Mann–Whitney test and Benjamini–Hochberg‐based FDR modes, *p < 0.05 and/or FDR < 0.05, compared with NDR.

First, partial least squares discriminant analysis (PLS‐DA) score plots were generated, and the permutation test showed that the models were reliable without overfitting (*R* ^2^ = (0.0, 0.12), *Q* ^2^ = (0.0, −0.12); *R* ^2^ = (0.0, 0.12), *Q* ^2^ = (0.0, −0.13); *R* ^2^ = (0.0, 0.06), and *Q* ^2^ = (0.0, −0.06) for DR versus NDR) (Figure S2A–C, Supporting Information) for GC‐MSM (**Figure** **2**A), LC‐MSM (Figure 2B), and LC‐MSL (Figure 2C) in the discovery set. The apparent separation of DR subjects from NDR subjects implied that abnormal metabolism occurred in DR, and a univariate analysis (nonparametric test) was subsequently performed for the identified metabolites from the three platforms between the DR and NDR groups. A total of 348 unique metabolites (93, 119, and 194 metabolites from GC‐MSM, LC‐MSM, and LC‐MSL, respectively) met the criteria of *p* < 0.05 and false‐discovery rate (FDR) < 0.05, showing significant changes between the DR and NDR groups (Figure 2D). Additionally, the nonparametric test was also used for lipids, and multiple lipid (sub)classes (e.g., fatty acids, phospholipids, and sphingolipids) increased significantly in the DR subjects compared to the NDR subjects (Figure 2E).

### 2.2 Metabolic and Lipid Profiling of Early‐Stage DR (NPDR)

The detection of early‐stage DR is very important for treatment. Thus, samples of early‐stage DR, namely NPDR, were enrolled to investigate metabolic and lipid profiles. The PLS‐DA score plots without overfitting (Figure S2D–F, Supporting Information) show that the NPDR group was also obviously detached from the NDR group based on metabolomic data from GC‐MSM (Figure 2H), LC‐MSM (Figure 2I), and LC‐MSL (Figure 2J). In addition, a total of 81, 103, and 156 metabolites from the GC‐MSM, LC‐MSM, and LC‐MSL platforms, were significantly different with *p* < 0.05 and FDR < 0.05 between the NPDR and NDR groups (Figure 2K). Additionally, the total levels of multiple lipid (sub)classes (e.g., fatty acids, phospholipids, and sphingolipids) displayed a significant increase in the NPDR subjects relative to the NDR subjects (Figure 2L).

### 2.3 Metabolic and Lipid Profiling of Different Clinical Grades of DR

Finally, nonparametric tests were performed on the basis of the identified metabolites with less than 30% RSD in the discovery study to identify the differential metabolites between two clinical grades of NDR, NPDR, MNPDR, SNPDR, and PDR. The numbers of significantly differential metabolites with *p* < 0.05 and/or FDR < 0.05 are listed in Table S1, Supporting Information. A large number of metabolites showed significant changes in the PDR and non‐PDR (including NPDR, MNPDR, and SNPDR) groups compared with the NDR group on the three analytical platforms. Some metabolites displayed a significant alteration in the PDR group compared to the non‐PDR group. However, few metabolites showed a significant change in pairwise comparisons among NPDR, MNPDR, and SNPDR. Subsequently, molecular trajectory plots were generated, and are shown in Figure S3, Supporting Information, based on metabolomic data from GC‐MSM, LC‐MSM, and LC‐MSL. Briefly, the NDR group is apparently separated from the other groups (e.g., NPDR, MNPDR, SNPDR, and PDR) in Figure S3, Supporting Information. Additionally, we also observed a tendency for the PDR group to move closer to the NDR group, implying that the PDR group shares more similar metabolic molecular characteristics to the NDR group than the other groups (e.g., NPDR, MNPDR, and SNPDR).

### 2.4 Differential Pathway Analyses

To systematically evaluate the perturbed metabolism underlying DR development, we performed pathway analyses based on metabolomics data from the discovery cohort. DR‐induced metabolic disturbances were mainly associated with glycolysis metabolism, TCA metabolism, urea cycle metabolism, polyol metabolism, amino acid metabolism (e.g., glycine, serine, and threonine metabolism; taurine, and hypotaurine metabolism; arginine–proline metabolism; valine–leucine–isoleucine biosynthesis), and lipid metabolism (e.g., phospholipid metabolism, sphingolipid metabolism, glyceride metabolism, and fatty acid metabolism).

### 2.5 Metabolite Markers and Diagnostic Performance for DR in the Discovery Set

Among the significantly altered metabolites in the discovery set, the levels of 12‐HETE and 2‐piperidone were much higher in the DR subjects than in the NDR subjects (Figure 2F). A binary logical regression analysis was carried out for the DR and NDR groups based on 12‐HETE and 2‐piperidone to produce a biomarker panel. The diagnostic performance of the biomarker panel (AUC 0.946) was better than that of 12‐HETE (AUC 0.924), 2‐piperidone (AUC 0.882), and HbA1c (AUC 0.691) in discriminating DR from NDR (**Table** **1**). Moreover, the biomarker panel showed higher sensitivity and specificity (0.894 and 0.919) than HbA1c (0.657 and 0.686) (Table 1).

**Table 1:** Results of different metabolite markers and HbA1c for the diagnosis of DR

Groups*	Metabolite Panel	Discovery Set (461)			Validation set (444)		
		AUC	Sensitivity	Specificity	AUC	Sensitivity	Specificity
		[95% Cl]	[%]	[%]	[95% Cl]	[%]	[%]
NDR versus DR	2‐piperidone	0.882	0.760	0.937	0.762	0.764	0.695
		(0.849–0.914)			(0.709–0.815)		
NDR versus NPDR		0.893	0.788	0.937	0.760	0.748	0.695
		(0.846–0.941)			(0.695–0.825)		
NDR versus MNPDR		0.903	0.856	0.865	0.724	0.699	0.705
		(0.856–0.949)			(0.655–0.793)		
NDR versus SNPDR		0.905	0.835	0.955	0.819	0.885	0.676
		(0.854–0.956)			(0.763–0.875)		
NDR versus PDR		0.815	0.684	0.946	0.649	0.700	0.676
		(0.746–0.884)			(0.487–0.810)		
NDR versus DR	12‐HETE	0.924	0.869	0.883	0.897	0.785	0.914
		(0.895–0.952)			(0.866–0.929)		
NDR versus NPDR		0.923	0.919	0.856	0.910	0.845	0.914
		(0.886–0.961)			(0.868–0.951)		
NDR versus MNPDR		0.940	0.889	0.865	0.862	0.709	0.914
		(0.908–0.971)			(0.811–0.912)		
NDR versus SNPDR		0.958	0.953	0.883	0.921	0.885	0.867
		(0.930–0.985)			(0.883–0.958)		
NDR versus PDR		0.867	0.763	0.883	0.888	0.850	0.867
		(0.812–0.922)			(0.791–0.985)		
NDR versus DR	Panel	0.946	0.894	0.919	0.928	0.805	0.933
		(0.921–0.970)			(0.900–0.956)		
NDR versus NPDR		0.958	0.929	0.901	0.925	0.816	0.933
		(0.930‐0.986)			(0.888‐0.962)		
NDR versus MNPDR		0.948	0.911	0.901	0.862	0.835	0.857
		(0.915–0.981)			(0.811–0.912)		
NDR versus SNPDR		0.980	0.929	0.964	0.952	0.867	0.905
		(0.961–0.999)			(0.926–0.977)		
NDR versus PDR		0.876	0.750	0.946	0.914	0.950	0.905
		(0.820–0.931)			(0.818–1.000)		
NDR versus DR	HbA1c	0.691	0.657	0.686	0.555	0.392	0.760
		(0.634–0.749)			(0.494–0.615)		
NDR versus NPDR		0.648	0.611	0.686	0.518	0.327	0.760
		(0.572–0.725)			(0.438–0.597)		
NDR versus MNPDR		0.715	0.596	0.781	0.610	0.495	0.760
		(0.643–0.787)			(0.532–0.688)		
NDR versus SNPDR		0.779	0.766	0.695	0.572	0.409	0.760
		(0.707–0.850)			(0.495–0.649)		
NDR versus PDR		0.627	0.644	0.638	0.544	0.700	0.423
		(0.538–0.717)			(0.411–0.678)		

*Panel: 12‐HETE & 2‐piperidone

It was also observed that the levels of 12‐HETE and 2‐piperidone were higher in the NPDR group than in the NDR group (Figure 2M). The diagnostic performance of the biomarker panel (AUC 0.958) was better than that of 12‐HETE (AUC 0.923), 2‐piperidone (AUC 0.893), and HbA1c (AUC 0.648) in the discrimination of NPDR from NDR (Table 1). Furthermore, the biomarker panel showed higher sensitivity and specificity (0.929 and 0.901) than HbA1c (0.611 and 0.686) in differentiating NPDR from NDR (Table 1). These results highlight the early diagnostic potential of this metabolite biomarker panel.

### 2.6 Metabolite Markers and Diagnostic Performance for DR in the Validation Set

To validate the diagnostic performance of the biomarker panel in distinguishing DR or early‐stage DR from NDR, we studied another independent cohort (validation set) with 444 samples (including 105 NDR, 103 NPDR, 103 MNPDR, 113 SNPDR, and 20 PDR samples) (**Table** **2**). 12‐HETE and 2‐piperidone were quantified in the multiple reaction monitoring (MRM) mode (Table S2, Supporting Information) based on the LC‐MS platform.

**Table 2:** Clinic characteristics of the subjects in each set

Characteristics	Discovery set (461)					Validation set (444)				
	NDR	NPDR	MNPDR	SNPDR	PDR	NDR	NPDR	MNPDR	SNPDR	PDR
N	111	99	90	85	76	105	103	103	113	20
Age [years]	63.24 ± 6.13	63.32 ± 6.06	63.03 ± 6.6	62.31 ± 6.14	63.17 ± 6.45	61.99 ± 7.50	62.68 ± 9.89	65.96 ± 9.03**	64.76 ± 9.88	70.95 ± 8.98***^##^
Sex [male/female]	50/61	48/51	40/50	32/53	31/45	59/46	37/66*	33/70**	46/67	13/7^a^
BMI [kg m^−2^]	25.41 ± 3.25	25.57 ± 3.37	25.39 ± 3.07	24.89 ± 3.34	24.98 ± 3.32	26.31 ± 3.24	25.02 ± 2.96*	26.22 ± 2.99^#^	25.51 ± 3.06	24.66 ± 2.77
DBP1 [mmHg]	81.55 ± 8.69	82.46 ± 10.51	81.83 ± 10.97	82.24 ± 9.52	79.33 ± 10.20	86.62 ± 11.54	83.68 ± 12.54	81.71 ± 11.61*	82.78 ± 10.87	83.25 ± 12.87
SBP1 [mmHg]	136.11 ± 17.60	144.03 ± 19.49*	145.04 ± 19.11**	145.90 ± 21.35*	144.03 ± 20.60*	145.2 ± 17.83	146.43 ± 20.21	146.89 ± 23.4	148.61 ± 18.71	144.5 ± 20.3
T2DM duration [years]	8.07 ± 5.56	7.46 ± 4.39	9.41 ± 4.90	11.44 ± 5.55	12.02 ± 6.08	8.2 ± 6.07	6.35 ± 4.74	8.21 ± 5.53	8.35 ± 6.53	6.41 ± 4.65
FPG [mmol.L^−1^]	8.32 ± 1.93	8.51 ± 1.95	9.02 ± 2.09	9.60 ± 2.09**^#^	8.06 ± 2.16ª^bbb^	8.97 ± 2.96	8.12 ± 2.96	9.76 ± 3.20^##^	9.13 ± 3.23	8.67 ± 2.50
HbA1c [%]	7.07 ± 1.23	7.77 ± 1.36**	7.97 ± 1.33***	8.49 ± 1.31***^#^	7.77 ± 1.37**^b^	7.33 ± 1.44	7.13 ± 1.94	8.03 ± 1.87*^#^	7.76 ± 1.93	7.46 ± 1.55
TC [mmol L^−1^]	5.14 ± 1.05	5.15 ± 1.05	5.22 ± 1.14	5.09 ± 1.12	5.33 ± 1.01	5.47 ± 2.24	4.92 ± 1.35	5.2 ± 1.21	5.3 ± 1.31	5.97 ± 1.3^#a^
TG [mmol L^−1^]	1.52 ± 0.72	1.48 ± 0.74	1.54 ± 0.71	1.36 ± 0.59	1.48 ± 0.79	2.39 ± 5.22	1.53 ± 0.77	1.68 ± 1.13	1.83 ± 1.53	1.71 ± 0.81
HDL‐c [mmol L^−1^]	1.32 ± 0.30	1.41 ± 0.34	1.44 ± 0.34	1.41 ± 0.35	1.37 ± 0.35	1.31 ± 0.33	1.38 ± 0.49	1.57 ± 1.16	1.43 ± 0.50	1.63 ± 0.53
LDL‐c [mmol L^−1^]	3.04 ± 0.95	3.10 ± 0.90	3.15 ± 0.99	2.96 ± 1.00	3.20 ± 0.88	3.14 ± 0.98	3.02 ± 1.09	3.01 ± 1.01	3.14 ± 0.96	3.6 ± 0.93
HGB [g L^−1^]	140.83 ± 13.35	146.08 ± 14.92	141.49 ± 14.09	139.30 ± 14.68^#^	137.08 ± 13.80^###^	142.7 ± 14.52	138.62 ± 29.07	139.74 ± 15.44	140.28 ± 22.06	145.89 ± 22.10
MCHC [g L^−1^]	331.58 ± 9.77	335.84 ± 11.52*	337.19 ± 10.69**	335.46 ± 15.09	334.98 ± 11.22	331.45 ± 13.01	323.93 ± 62.83	330.42 ± 33.6	330.97 ± 37.53	336.21 ± 9.98
MCH [pg]	30.25 ± 1.32	30.28 ± 1.51	30.08 ± 1.46	29.97 ± 1.66	29.95 ± 1.26	30.33 ± 1.81	29.36 ± 5.52	30.16 ± 1.73	29.95 ± 3.49	30.11 ± 1.75
MCV [fl]	91.20 ± 3.61	90.09 ± 4.42	89.27 ± 4.18*	89.72 ± 4.46	89.77 ± 3.99	91.53 ± 4	87.85 ± 16.41	90.65 ± 4.9	89.85 ± 10.41	89.78 ± 4.88
PLT [10^9^ L^−1^]	188.13 ± 62.30	198.20 ± 52.99	183.72 ± 50.96	194.88 ± 52.94	196.06 ± 62.75	193.28 ± 54.85	182.82 ± 66.32	187.78 ± 60.02	206.03 ± 82.42	212.7 ± 44.6
RBC [10^12^ L^−1^]	4.69 ± 0.47	4.84 ± 0.47	4.72 ± 0.45	4.66 ± 0.50	4.58 ± 0.48^#^	4.7 ± 0.46	4.59 ± 0.92	4.66 ± 0.53	4.7 ± 0.68	4.86 ± 0.75
WBC [10^9^ L^−1^]	6.25 ± 1.60	6.40 ± 1.32	6.18 ± 1.52	6.56 ± 1.44	6.76 ± 1.63	6.22 ± 1.47	6.52 ± 2.31	6.28 ± 1.79	6.73 ± 1.9	6.72 ± 1.07
Urea [mmol L^−1^]	5.99 ± 1.44	5.98 ± 1.37	6.16 ± 1.35	6.36 ± 1.54	6.05 ± 1.30	5.92 ± 1.71	5.5 ± 1.77	13.6 ± 71.6^#^	6.06 ± 1.78	6.11 ± 1.22
UricAcid [µmol L^−1^]	320.27 ± 74.47	308.45 ± 75.42	306.39 ± 85.18	315.58 ± 85.07	306.72 ± 84.33	320.6 ± 86.69	269.39 ± 144.58*	256.27 ± 147.2*	268.76 ± 128.84	244.34 ± 149.3
ALP [U L^−1^]	80.81 ± 21.90	81.21 ± 22.00	76.18 ± 21.78	82.82 ± 19.73	81.83 ± 23.61	85.04 ± 22.42	80.33 ± 27.64	82.62 ± 20.49	84.04 ± 29.24	86.35 ± 26.01
ALT [U L^−1^]	20.528.39	20.207.86	20.288.60	20.15 ± 8.76	18.37 ± 8.99	24.19 ± 13.5	21.99 ± 17.22	20 ± 10.35	21.21 ± 15.64	21.45 ± 13.12
AST[U L^−1^]	22.02 ± 4.97	20.11 ± 4.08*	19.75 ± 5.47*	21.22 ± 5.66	19.76 ± 4.90*	25.41 ± 12.84	22.14 ± 10.45*	21.79 ± 8.23*	22.71 ± 17.4**	21.15 ± 7.58
DBILI [µmol L^−1^]	3.95 ± 1.46	3.31 ± 1.41*	3.01 ± 1.44***	3.18 ± 1.37*	3.05 ± 1.26**	4.36 ± 2.01	3.84 ± 2.36	3.4 ± 1.95***	3.69 ± 2.19**	2.91 ± 0.90**
HCT [%]	42.44 ± 3.63	43.30 ± 4.14	41.61 ± 3.85	40.80 ± 4.67^#^	40.56 ± 4.49^#^	41.8 ± 8.00	41.39 ± 8.81	41.47 ± 6.23	41.75 ± 6.48	43.34 ± 6.03
TBIL [µmol L^−1^]	13.70 ± 4.37	13.82 ± 4.44	13.76 ± 4.29	11.58 ± 3.90*^###^ª	11.35 ± 4.00*^#^ª	15.21 ± 5.3	13.76 ± 6.22	13.99 ± 5.18	13.77 ± 6.12*	13.74 ± 6.2

The clinical data were compared using nonparametric tests in Wilcoxon, Mann–Whitney test model, *p < 0.01, **p < 0.001, ***p < 0.0001 when compared with NDR; ^#^ *p* < 0.01, ^##^ *p* < 0.001, ^###^ *p* < 0.0001 when compared with NPDR; ª*p* < 0.01, ªª*p* < 0.001, ªªª*p* < 0.0001 when compared with MNPDR; ^b^ *p* < 0.01, ^bb^ *p* < 0.001, ^bbb^ *p* < 0.0001 when compared with SNPDR. Data represent mean ± SD.

The serum concentrations of 12‐HETE and 2‐piperidone were much higher in DR and early‐stage DR than in NDR in the validation set (Figure 2G,N). These compounds were used to build a biomarker panel by means of a logistical regression model for DR and NPDR detection, and the constructed equations are as follows:

(1) $$\begin{array}{ccc} \mathrm{logit}[P & = & \mathrm{DR}]=0.165x\left[2-\mathrm{piperidone}\right]+0.037\\ & & \times \left[12-\mathrm{HETE}\right]-2.634 \end{array}$$

(2) $$\begin{array}{ccc} \mathrm{logit}\;[P & = & \;\mathrm{NPDR}]=\;0.124\;x\;\left[2-\mathrm{piperidone}\right]+0.027\\ & & \times \;\left[12-\mathrm{HETE}\right]-3.094 \end{array}$$

where [P = DR] and [P = NPDR] are the prediction probabilities of DR and NPDR with this panel, respectively, and [12‐HETE] and [2‐piperidone] are the absolute serum concentrations of 12‐HETE and 2‐piperidone, respectively. The units of absolute concentration are µg mL^−1^ for 2‐piperidone and ng mL^−1^ for 12‐HETE. The cut‐off values of [P = DR] and [P = NPDR] were 0.789 and 0.469, respectively. Similar to the results in the discovery set, the biomarker panel showed higher diagnostic performance in discriminating DR and NPDR from NDR than HBA1c. The corresponding parameters were (AUC 0.928, sensitivity 0.805, and specificity 0.933) versus (AUC 0.555, sensitivity 0.392, and specificity 0.760) for DR and NDR, respectively, and (AUC 0.925, sensitivity 0.816, and specificity 0.933) versus (AUC 0.518, sensitivity 0.327, and specificity 0.760) for NPDR and NDR, respectively (Table 1). We can see from Table 1 and above data that although HbA1c is the only validated systemic biomarker for DR progression,^[^ ^5^ ^]^ it is not a good enough to distinguish DR from NDR, in reverse, our newly defined metabolic marker panel can achieve better AUC for both discovery and validation groups.

### 2.7 Disorders of Metabolic and Lipid Pathways

**Figure 3:**
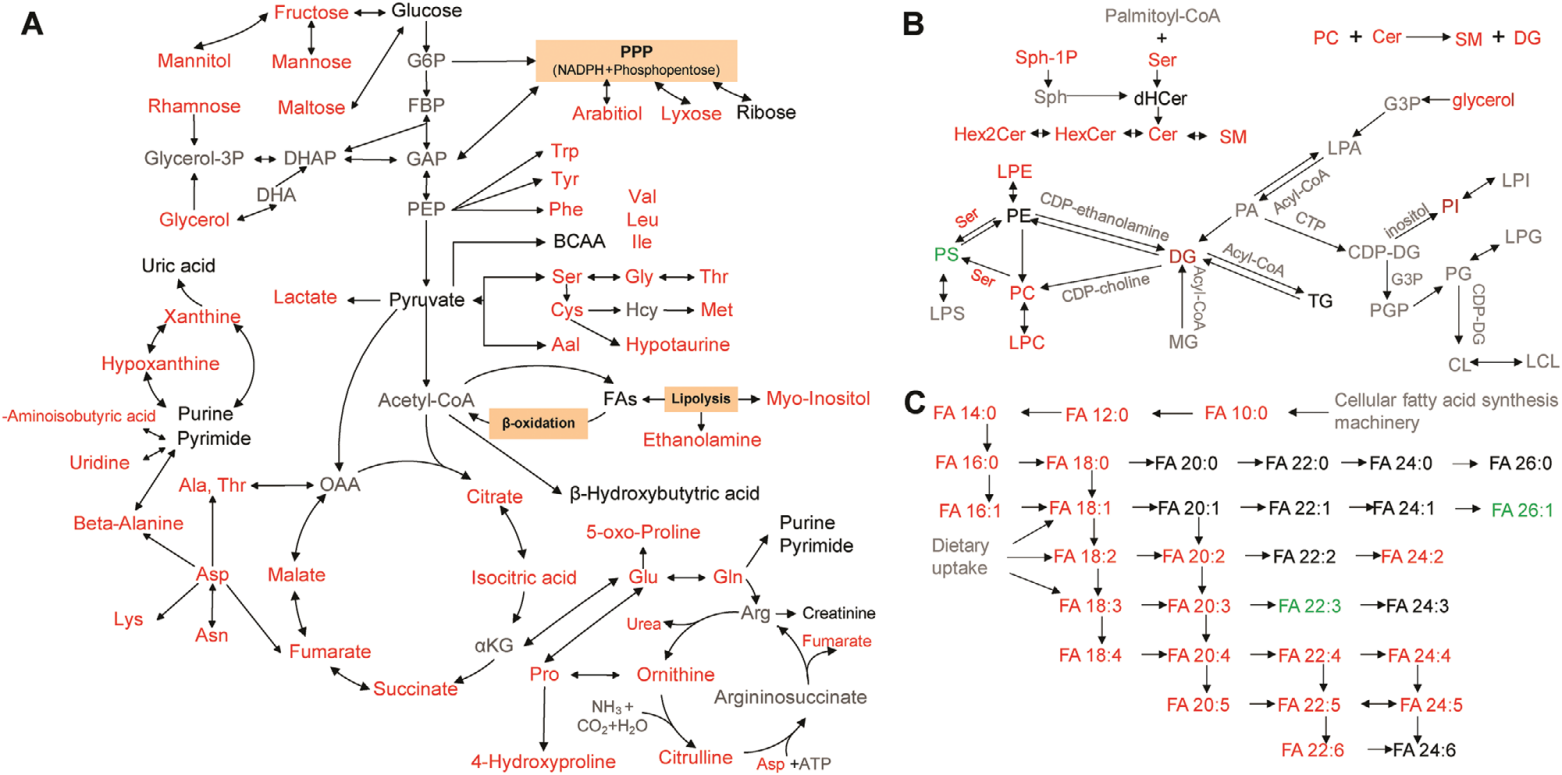
Changes in metabolic pathways in DR. Black text represents the detected metabolites with no significant change, red text represents significantly enriched metabolites (e.g., multiple amino acids), and green text represents significantly depleted metabolites (e.g., phosphatidylserine, PS) when the DR group is compared with the NDR group, and gray text represents undetected metabolites. The metabolite data were compared using nonparametric tests in Wilcoxon, Mann–Whitney test and Benjamini–Hochberg‐based FDR modes.

Metabolomics offers unique insights into disease pathways, as metabolites are the products of all biological processes, and their differential levels reflect the intricate interplay between environmental and genetic factors. Our study provides a systematic evaluation of serum metabolite profile changes associated with DR in individuals using multiple analytical platforms (GC‐MSM, LC‐MSM, and LC‐MSL) in order to cover as many metabolites as possible. Among the 348 unique metabolites described, we identified far more metabolites associated with the pathogenesis of DR than other studies, covering metabolites involved in TCA and urea cycle metabolism, (branched‐chain) amino acids, polyhydric alcohols, nucleotides, and their derivatives, carnitines, bile acids, and lipids, etc. (Table S3–S5, Supporting Information). Moreover, complete metabolic pathway analysis using discriminating metabolites was performed to explore pathway‐based metabolomic features, which are mainly associated with energy metabolism, amino acid metabolism, and lipid metabolism (**Figure** **3**).

Energy metabolism plays a key role in DR onset and development. Glycolysis and the TCA cycle, as two major energy metabolism pathways, caught our attention. We found that lactate which is related to glucose metabolism showed a significant increase in DR patients (Figure 3A). Additionally, the levels of citrate, isocitrate, succinate, fumarate, and malate were also significantly increased in DR compared to NDR (Figure 3A; Table S3, Supporting Information), reflecting the possibility of an increase in the TCA system. Many studies^[^ ^12^ ^]^ have indicated that mitochondrial dysfunction is closely related to DM and its complications. We observed that the fold changes of metabolites on the right side (fumarate and malic acid) were less than those on the left side (citric acid, isocitric acid, and succinic acid) in the TCA pathway in DR relative to NDR. We assumed that the intermediates on the right side were used to enhance the urea cycle, and the intermediates on the left side were replenished from aspartic acid metabolism. The increase in aspartic acid metabolism and the urea cycle in DR seem to confirm the above hypothesis; however, the detailed mechanism should be further studied. Overall, increased energy metabolism is associated with DR onset and progression.

We found that the levels of most amino acids were significantly increased in DR compared with NDR (Figure 3A; Table S3–S4, Supporting Information). The serine and glycine residues could enrich the photoreceptor protein rhodopsin, which is closely associated with the phosphorylation of pyruvate kinase M2, and consequently stabilize the Warburg‐like effect.^[^ ^13^ ^]^ The decrease in rhodopsin is a result of the relative vitamin A deficiency in ocular tissues in diabetes.^[^ ^14^ ^]^ The high levels of arginine, ornithine, citrulline, and proline in DR^[^ ^15^ ^]^ reflect the upregulation of arginine metabolism. Consistent with many other studies,^[^ ^16^ ^]^ the increase in arginine metabolism could be a mediator of DR, and the levels of leucine, isoleucine, and valine in branched‐chain amino acid (BCAA) metabolism were also increased. The increasing levels of circulating BCAAs are considered to be related to the intense neurotoxicity of glutamate in the retina, which plays a major role in DR neurodegeneration.^[^ ^17^ ^]^ Fundamentally, BCAAs exert their effect by activating the mammalian target of rapamycin (mTOR) pathway, which functions in the regulation of cell growth, proliferation, and survival and in upregulation of the VEGF pathway. Activation of the VEGF pathway leads to increased expression of Caspase‐3, which consequently causes retinal damage.^[^ ^18^ ^]^ Thus, we think that more attention should be paid to the levels of amino acids in circulation, which would contribute to understanding the pathogenesis of DR.

Moreover, the levels of most lipids displayed significant increases in DR relative to NDR. Sphingolipids, as one of the essential components of lipids, have gained increasing attention in recent years due to their key roles in signal transduction, cell proliferation, migration, apoptosis, and membrane structural components.^[^ ^19^ ^]^ According to recent evidence, ceramide, sphingosine, and sphingosine‐1‐phosphate (S1P) have been identified as bioactive sphingolipids,^[^ ^20^ ^]^ and S1P influences vascular formation, differentiation, and endothelial cell migration.^[^ ^21^ ^]^ Alterations in the sphingolipid profile, such as transformation of individual molecules of ceramide and certain hexose‐ceramides, have been reported in diabetes and diabetes‐induced DR.^[^ ^22^ ^]^ Therefore, a high level of sphingolipids derived from increased phosphatidylcholine and sphingolipid biosynthesis (Figure 3B) may aggravate cell proliferation and blood vessel formation, playing a very important role in DR onset and progression.

### 2.8 Identification and Validation of Biomarkers

Currently, it is still an important goal to identify novel potential serum biomarkers for detecting DR. After systematic screenings utilizing univariate analyses, a biomarker panel including 2‐piperidone and 12‐HETE was identified and validated. Early screening of DR remains a challenge. In this study, the early diagnostic performance of the serum metabolite panel was also tested in patients with NPDR. This panel showed very good performance in separating DR and early‐stage DR (NPDR) from a high‐risk population (NDR). The diagnostic accuracy ranges of this panel for DR were 75.0–92.9% and 80.6–95.0% in the discovery and validation sets, respectively (Figure S4, Supporting Information).

12‐HETE, an eicosanoid, is the main product of 12‐lipoxygenase (LOX) in humans and induces endoplasmic reticulum (ER) stress in hRECs. Many studies have shown that disordered eicosanoid metabolism plays a crucial role in disease progression. We found that circulating 12‐HETE levels increased progressively in DR and were positively associated with the onset and progression of DR. Many studies have suggested that 12‐LOX participates in retinal microvascular dysregulation in DR by activating ER stress, NADPH oxidase, and the VEGFR2 signaling network, and that destruction of Ca^2+^ homeostasis may be a necessary step in promoting the signaling pathway (Figure S5, Supporting Information).^[^ ^23^ ^]^

The other biomarker, 2‐piperidone, also known as *δ*‐valerolactam, is a monomer that is widely used in industry to synthesize polymers (nylon‐5). However, the presence of 2‐piperidone in serum has not been reported to date. In our study, circulating 2‐piperidone levels increased greatly in DR and early‐stage DR. Some studies have reported that the increased level of 2‐piperidone may be caused by dietary intake and conversion of cadaverine, which is a general semen and urine metabolite produced by decarboxylation of lysine.^[^ ^24^ ^]^ Cheng et al. reported that 2‐piperidone is a potential metabolite biomarker of CYP2E1 activity because the conversion of cadaverine to 2‐piperidone and that of 2‐piperidone to 6‐hydroxy‐2‐piperidone were positively associated with CYP2E1.^[^ ^25^ ^]^ However, we could not find related pathways or biological functions of 2‐piperidone identified by cell biological investigation. Thus, we studied the biological functions of 2‐piperidone in hRECs to determine whether 2‐piperidone had a biological function associated with DR progression.

The viability of hRECs with 2‐piperidone at a concentration ≥0.4 µg mL^−1^ was significantly increased compared with that of the controls at 24 and 72 h (Figure S6 A,B, Supporting Information). 2‐Piperidone at over 0.4 µg mL^−1^ could promote hREC proliferation. The relative mRNA expression levels of VEGFA and VEGFR2 in hRECs treated with various concentrations (0.75, 1.5, 3 µg mL^−1^) of 2‐piperidone were obviously increased compared to the levels in the controls (Figure S6C, Supporting Information). In addition, the relative concentration of VEGF in the culture medium of hRECs treated with 2‐piperidone was significantly elevated compared to that in medium treated with the vehicle (Figure S6D, Supporting Information). The expression of VEGFR2 in hRECs treated with 2‐piperidone also increased significantly (Figure S6E, Supporting Information). Importantly, we observed obvious tube formation in hRECs treated with 2‐piperidone (Figure S6F,G, Supporting Information). These results seem to imply that 2‐piperidone was able to promote angiogenesis. The relative mRNA expression levels of related genes (e.g., TNF‐*α*, IL‐6, ICAM1, and VCAM1) in hRECs treated with 2‐piperidone at concentrations of 0.75, 1.5, and 3 µg mL^−1^ were significantly increased compared with those in the controls (Figure S6H, Supporting Information). Based on immunoblots of ICAM1 and VCAM1 (Figure S6I, Supporting Information) and the relative concentrations of IL‐6 and TNF‐α (Figure S6J,K, Supporting Information) in hRECs treated with 2‐piperidone, significant upregulation was observed, suggesting that 2‐piperidone was closely related to proinflammatory activity. The results showed that a novel biomarker, 2‐piperidone, independent of 12‐HETE (correlation coefficients 0.26 and 0.01 in the discovery and validation sets, respectively), could promote hREC proliferation, angiogenesis, and inflammation, which are closely related to DR development.

## 3 Conclusions

In summary, our aforementioned data highlight the potential importance of metabolomics studies for determining the pathogenesis of DR and suggest that metabolomics profiling could effectively identify diagnostic markers of DR and early‐stage DR from high‐risk Chinese populations. Moreover, our results showed that a novel biomarker (2‐piperidone) has biological functions in the promotion of hREC proliferation, angiogenesis, and inflammation. To the best of our knowledge, this is the most comprehensive metabolomics study with a large population investigating the association between metabolic profiles and DR onset and progression. This multiplatform‐based metabolomics study provides a practical strategy for comprehensively exploring intricate metabolic networks in DR and screening DR with a small amount of serum. The results could be utilized as a reference for further clinical examination.

## 4 Experimental Section

The clinical stages of DR were classified as NPDR, MNPDR, SNPDR, and PDR according to the Early Treatment Diabetic Retinopathy Study (ETDRS) grading system.^[^ ^26^ ^]^ The clinical grade of DR was diagnosed by professional ophthalmologists according to the ETDRS. NDR subjects were defined as a diabetic population without retinopathy, based on the World Health Organization guidelines.

A total of 905 subjects, including NDR and DR subjects from the Shanghai Integrated Diabetic Prevention and Care System Study, were enrolled in this project. The distributions of age and sex in the different clinical grades were matched as much as possible in the discovery and validation sets, and the detailed clinical characteristics of the subjects are provided in Table 2. In the discovery set, all 461 sex and age‐matched fasting serum samples, including 111 NDR, 99 NPDR, 90 MNPDR, 85 SNPDR, and 76 PDR samples, were collected and analyzed by three platforms (GC‐MSM and LC‐MSM and LC‐MSL) to comprehensively elucidate abnormal metabolites and intricate pathways of DR and identify serum biomarkers for the diagnosis of DR. A novel biomarker panel was tested in the validation cohort of 444 participants, containing 105 NDR, 103 NPDR, 103 MNPDR, 113 SNPDR, and 20 PDR cases. Finally, the influence of a novel marker, 2‐piperidone, on hRECs was investigated to understand the mechanism (Figure 1). The study was approved by the Ethical Committee of Shanghai Sixth People's Hospital (2018‐KY‐066 (K)) and conducted in accordance with the Declaration of Helsinki. Written informed consent was obtained from all participants before inclusion in the project.

##### Metabolomics and Lipidomics Analyses

After overnight fasting, all of the serum samples were collected and stored in a −80 °C freezer. Prior to analysis, the samples were thawed on ice. Details regarding sample pretreatment and metabolomics and lipidomics analyses based on GC‐MS and LC‐MS are provided in the Supporting Information.

##### Cell Biological Study of 2‐Piperidone Function

The description of the cell experiment with 2‐piperidone is also provided in the Supporting Information.

##### Statistical Analysis

First, the raw data from GC‐MSM, LC‐MSM, and LC‐MSL were normalized by corresponding internal standards to minimize errors arising from the sample pretreatment and analysis procedures as much as possible.

A supervised PLS‐DA model with unit variance scaling was carried out by SIMCA‐P 13.0 (Umetrics, Umeå, Sweden) software for the data from GC‐MSM, LC‐MSM, and LC‐MSL. To assess the overfitting risk of the model, a permutation test with 200 iterations was performed.

Univariate analysis was performed with the open‐source Multiple Experiment Viewer (MeV version 4.9.0 software) for the normalized metabolite data from GC‐MSM, LC‐MSM, and LC‐MSL. Nonparametric tests in Wilcoxon, Mann–Whitney test, and Benjamini–Hochberg‐based FDR modes were performed, setting *p* < 0.05 and/or FDR < 0.05 as the significant difference levels. Metabolite‐associated pathways were analyzed using MetaboAnalyst 4.0 (Xia Lab at McGill University, Montreal, Canada; metaboanalyst.ca).

A binary logistic regression analysis was employed to establish the model with differential metabolites to define biomarker candidates using IBM SPSS 19 software (SPSS, Inc.). The receiver‐operating characteristic curve was utilized to evaluate the results of the regression analysis.

For the clinical data of patients, all values were expressed as means ± standard deviations (SD).

## Conflict of Interest

The authors declare no conflict of interest.

## Author Contributions

Q.X.,Y.O.,Y.W., and L.W. contributed equally to this work. G.X., W.J., J.W., Q.X., Y.O., Y.W., L.W., and H.L. conceived and designed the study, interpreted the data, critically revised the manuscript for important intellectual content, and approved the version to be published. Q.X., Y.O., Y.W., Y.L., and D.F. contributed to data acquisition and statistical analyses. Q.X. contributed to writing the manuscript. X.Z., C.H., L.Z., X.L., W.Q., H.Z., and C.C. interpreted the data, critically revised the manuscript for important intellectual content and approved the version to be published. G.X., W.J., and J.W. are the guarantors of this work and, as such, had full access to all the data in the study and take responsibility for the integrity of the data and the accuracy of the data analysis

## Supporting information

Supporting Information
