# Supplementary material for: Multiplatform Metabolomics Reveals Novel Serum Metabolite Biomarkers in Diabetic Retinopathy Subjects
Source: Adv Sci (Weinh). 2020 Oct 1;7(22):2001714. doi: 10.1002/advs.202001714 (PMC7675050; doi:10.1002/advs.202001714)
Supplement: Supplementary file 1 — Supporting Information [file ADVS-7-2001714-s001.pdf]

© 2020 Wiley-VCH GmbH

ADVANCED SCIENCE
Open Access

Supporting Information

for *Adv. Sci.*, DOI: 10.1002/advs. 202001714

Multiplatform Metabolomics Reveals Novel Serum Metabolite Biomarkers in Diabetic Retinopathy Subjects

Qiuhui Xuan, Yang Ouyang, Yanfeng Wang, Liang Wu, Huating Li, Yuanyuan Luo, Xinjie Zhao, Disheng Feng, Wangshu Qin, Chunxiu Hu, Lina Zhou, Xinyu Liu, Haidong Zou, Chun Cai, Jiarui Wu, Weiping Jia,* and Guowang Xu**

Supplemental material

Multiplatform Metabolomics Reveals Novel Serum Metabolite Biomarkers in Diabetic Retinopathy Subjects

Qiuhui Xuan[1,2#], Yang Ouyang[1,2#], Yanfeng Wang[1,2#], Liang Wu[3#], Huating Li[3], Yuanyuan Luo[1,2], Xinjie Zhao[1], Disheng Feng[1,2], Wangshu Qin[1], Chunxiu Hu[1], Lina Zhou[1], Xinyu Liu[1], Haidong Zou[4], Chun Cai[3], Jiarui Wu[5]*, Weiping Jia[3*], Guowang Xu[1,2*]

[1] CAS Key Laboratory of Separation Science for Analytical Chemistry, Dalian Institute of Chemical Physics, Chinese Academy of Sciences, 457 Zhongshan Road, Dalian 116023, China.

[2] University of Chinese Academy of Sciences, Beijing 100049, China.

[3] Shanghai Diabetes Institute, Shanghai Key Laboratory of Diabetes Mellitus,Shanghai Clinical Center for Endocrine and Metabolic Diseases, Metabolic diseases biobank, Shanghai JiaoTong University Affiliated Sixth People's Hospital, Shanghai 200233,China.

[4] Department of Ophthalmology, First People's Hospital of Shanghai, Shanghai Jiao Tong University, Shanghai, China.

[5] Key Laboratory of Systems Biology, CAS Center for Excellence in Molecular Cell Science, Institute of Biochemistry and Cell Biology, University of Chinese Academy of Sciences, Chinese Academy of Sciences, 320 Yue-Yang Road, Shanghai 200031, China

*Corresponding authors:

Prof. Dr. Guowang Xu, CAS Key Laboratory of Separation Science for Analytical

Chemistry, Dalian Institute of Chemical Physics, Chinese Academy of Sciences, Dalian 116023, China. Tel. / Fax: 0086-411-84379530. E-mail: xugw@dicp.ac.cn.

Prof. Weiping Jia, Shanghai Diabetes Institute, Shanghai Key Laboratory of Diabetes Mellitus, Shanghai Clinical Center for Endocrine and Metabolic Diseases, Shanghai Jiaotong University Affiliated Sixth People's Hospital, Shanghai 200233, China. E-mail: wpjia@sjtu.edu.cn .

Prof. Jiarui Wu, Key Laboratory of Systems Biology, CAS Center for Excellence in Molecular Cell Science, Institute of Biochemistry and Cell Biology, University of Chinese Academy of Sciences, Chinese Academy of Sciences, 320 Yue-Yang Road, Shanghai 200031, China. E-mail: wujr@sibs.ac.cn.

Metabolomic methods

Nontargeted GC-MS-based Metabolic Profiling

Samples were prepared and analyzed as previously described in our laboratory with some modification. Briefly, samples were thawed at 4 °C, and then, 50 μL of each serum sample was extracted with 200 μL of cold methanol (including valine-d8, succinic acid-d4, phenylalanine-d5 and tridecanoic acid at 5 μg/mL). The mixture was vortexed, and centrifuged (14000 rpm, *4 °C, *10 min), and then, the supernatant was lyophilized with a refrigerated centrifugal vacuum concentrator (CentriVap, Labconco, USA). The dried extract was derivatized with 50 μL of methoxyamine hydrochloride solution and 40 μL of N-methyl-N-(trimethylsilyl) trifluoroacetamide (MSTFA) prior to GC-MS analysis. A quality control (QC) sample was obtained by mixing an equal volume of serum samples and pretreated as described above.

GC-MS analysis was carried out via a GC-MS QP2010 Plus (Shimadzu., Japan), coupled with a DB-5MS capillary column (30 m × 0.25 mm id, 0.25 μm film thickness; Agilent Technologies, Santa Clara, CA, USA). The injected volume was 1 μL with a split ratio of 1:10, and the injection temperature was set as 300 °C. The initial column temperature was set at 80 °C and held for 1 min, linearly ramped to 210 °C at a rate of 30 °C/min, and then linearly raised to a final temperature of 320 °C at a rate of 20 °C/min and held for 4 min. The temperatures of the ion source and transfer line were set at 230 °C and 320 °C, respectively. The detection voltage value was set with reference to the tuning file. The data were acquired in full scan mode (m/z 33 - 600) with a scan speed of 0.20 s/scan, and the initial data acquisition time was 2.92 min. QC samples inserted into the analysis sequence every 10 samples were analyzed to evaluate the analytical quality.

Nontargeted LC-MS-based Metabolic Profiling

Each serum sample was extracted as previously described in our laboratory. In brief, 50 μL of serum sample from each subject was used for analysis. The serum was added to 200 μL of methanol and the mixture was mixed in 96-well plates. After vortexing, the extract precipitated in the 96-well plates and the protein was removed by centrifugation (4 °C, 10 min, 500 × *g*). Then, the extract was lyophilized and stored at -80 °C.

Before analysis, each sample was reconstituted with water/methanol (80:20, v/v). After vortexing and centrifugation, the 96-well plates were transferred into a liquid chromatography–mass spectrometry (LC-MS) system for analysis. Pooled QC samples were pretreated following the same procedure as described above. The QC samples were processed and detected after every ten actual serum samples.

A Waters ACQUITY UPLC system (Waters Corp, Milford, MA, USA) coupled with a Q Exactive HF mass spectrometer (Thermo Fisher Scientific, Bremen, Germany) was used for separation and metabolic analysis. Briefly, the injection volume was 5 μL, the column temperature was set to 60 °C, and the flow rate was 0.4 mL/min. In positive ion mode, the chromatography column used was a UPLC C8 column. Water and acetonitrile (both containing 0.1% formic acid) were utilized as mobile phases A and B, respectively. The starting composition was 5% B, which was maintained for 0.5 min before being increased to 40% at 2.0 min and 100% at 8.0 min for a 2.0 min wash, followed by returning to 5% B in 0.1 min and held until 12 min for a re-equilibration step. In negative ion mode, the chromatography column used was a UPLC T3 column. Water and H_2O/CH_3OH (5:95, v/v) (both containing 6.5 mmol/L NH_4HCO_3) were used as mobile phases A and B, respectively. The elution gradient was similar to that in the positive ion mode, except for the starting composition (2% B) and re-equilibration composition (2% B).

For metabolite analysis, the ion spray voltages were 3.5 and 3.0 kV in positive and negative ion modes, respectively. The full MS scan range was m/z 70–1050 with a resolution setting of 120,000 (FWHM) at m/z 200. The 10 most intense peaks were selected in data-dependent acquisition mode. The resolution setting for tandem mass spectra was 30,000 (FWHM) at m/z 200. The capillary temperature and aux gas heater temperature were 300 °C and 350 °C, respectively. The aux gas and sheath gas were set as 10 psi and 45 psi, respectively. The S-lens RF level was 50. The MS1 automatic gain control (AGC) target value was 3×10^6, and the maximum injection time was 100 ms. For MS/MS scans, the AGC target value was 1×10^5, and the maximum injection time was 50 ms.

Nontargeted LC-MS-based Lipidomics

Each serum sample was processed for lipid extraction by the MTBE/MeOH/H_2O system. Briefly, 300 μL of MeOH (containing PC 19:0/19:0 at 0.67 μg/mL, LPC 19:0 at 0.33 μg/mL, PE 17:0/17:0 at 0.33 μg/mL, SM d18:1/12:0 at 0.17 μg/mL, Cer d18:1/17:0 at 0.17 μg/mL, TG 15:0/15:0/15:0 at 0.53 μg/mL, FA 18:0_d3 at 0.67 μg/mL and FA 16:0_d3 at 0.67 μg/mL) was added to 40 μL of sample matrix followed by the addition of 1 mL of MTBE. Then, the mixture was vibrated for 10 min. Subsequently, 300 μL of H_2O was added and vortexed to form a two-phase system. Then, the mixture was centrifuged (14,000 rpm, 10 min, 4 °C). Two 400-μL aliquots of supernatant were lyophilized and stored at -80 °C prior to the lipidomic analysis. Lyophilized samples were reconstituted in ACN/IPA/H_2O (65:30:5, v/v/v/) containing 5 mM ammonium acetate, and 5 μL of the sample was injected into the LC-MS system.

An equal aliquot of serum from a large number of serum samples from the biobank in our laboratory was mixed to form pooled QC samples. The pretreatment of QC samples

was the same as that mentioned above. The QC samples were evenly inserted per 10 actual samples in the analysis batch to monitor the stability of the analysis.

A Waters ACQUITY UHPLC (Milford, U.S.A.) coupled to an AB SCIEX Triple Q-TOF 5600 Plus (Concord, Canada) was used for lipidomics analyses. A Waters BEH C8 column (2.1 mm x 100 mm, 1.7 μm) was used for lipid separation. The mobile phases consisted of 3:2 (v/v) ACN/H_2O (10 mM AcAm, phase A) and 9:1 (v/v) IPA/ACN (10 mM AcAm, phase B). The flow rate was set as 0.3 mL/min and the column temperature was 60 °C. The elution gradient started at 50% B, was held at this concentration for 1.5 min, was linearly increased to 85% B at 9.0 min, reached 100% B at 9.1 min, and was held at this concentration for 1.9 min. Finally, the column was returned to 50% B within 0.1 min and held at this concentration for 1.9 min for equilibration. The total run time was 13 min.

The ion spray voltage for MS was set at 5500 V and 4500 V in positive and negative ion modes, respectively. The interface heater temperature was 500 °C and 550 °C in positive and negative ion modes, respectively. Ion source gas 1, ion source gas 2, and curtain gas were set at 50, 50, and 35 psi in positive ion mode and 55, 55, and 35 psi in negative ion mode, respectively. The MS scan range was 300-1250 Da in positive mode and 150-1250 Da in negative mode.

Targeted LC-MS Analysis

In this study, a new targeted method was developed for quantitative analysis of 2-piperidone and 12-HETE. The 444 serum samples of the validation set were randomly ordered. The sample pretreatment was performed on an Impact 96-well protein precipitation plate (Phenomenex, Torrance, CA, USA). Forty microliters of serum were taken from each sample and 160 μL of methanol containing internal standards (phe-d5

and 12-HETE-d8 at concentrations of 0.2 μg/mL and 0.1 μg/mL, respectively) was added. Then the mixtures were mixed in the 96-well plates. Removal of proteins was achieved by centrifugation (4 °C, 10 min, 500 × *g*). The obtained filtrate was lyophilized in vacuo and stored at -80 °C. Before analysis, each sample was reconstituted in 120 μL of water/methanol (80:20, v/v). After vortexing and centrifugation (10 °C, 15 min, 800 × *g*), the 96-well plate was transferred to an LC-MS system for analysis. The QC samples were obtained by mixing equal volumes of serum samples randomly selected from the validation set. The QC samples were pretreated in the same way as other actual samples. Then the QC samples were analyzed after every 10 samples to evaluate the stability of the entire analysis process.

A Waters ACQUITY UPLC I-Class system (Waters Corp, Milford, MA, USA) was used. The column temperature was 40 °C, and the temperature of the sample compartment was 6 °C. The injection volume was 5 μL, and the flow rate was 0.4 mL/min. The column used was an ACQUITY UPLC BEH C8 column (2.1 mm× 50 mm, 1.7 μm particle size, Waters Corp., Milford, MA, USA). Mobile phase A was water containing 0.1% (v/v) formic acid and 2 mM ammonium acetate, and phase B was methanol containing 0.1% (v/v) formic acid and 2 mM ammonium acetate. The strong wash and weak wash solvents were methanol/water (90:10, v/v) and methanol/water (10:90, v/v), respectively. The initial gradient was 20% B, which was maintained for 0.2 min, linearly increased to 100% B at 0.8 min, maintained for 1.7 min, and returned to the initial value in the next 0.1 min. The initial gradient was then used to equilibrate the column until 4 min.

A Waters Xevo TQ-XS mass spectrometer was used. Standard samples of 2-piperidone, phe-d5, 12-HETE, and 12-HETE-d8 were injected separately and the cone voltage and collision energy were optimized (Table S1). Initially, the mass spectrometer was

operated in electrospray positive ion mode and switched to negative ion mode at 1 min. Then, the positive ion detection mode was used at 2 min for equilibrium held for 2 minutes.

Cell experiment

Cell Culture

Human retinal endothelial cells (hRECs) were purchased from the BeNa Culture Collection and maintained in Dulbecco's modified Eagle's medium (DMEM, Gibco) supplemented with 10% fetal bovine serum (BI) and incubated in an atmosphere incubator containing 5% CO_2 at 37 °C. For 2-piperidone treatment, hRECs were maintained in 2% DMEM overnight (serum starvation) at approximately 80% confluency and then treated with PBS control or 2-piperidone in complete medium.

CCK8

The CCK8 assay was used to examine cell proliferation. After serum starvation, hRECs (1×10^3) were seeded in 96-well plates in a volume of 100 µL. After attachment, the cells were exposed to different doses of 2-piperidone (0, 0.05, 0.1, 0.2, 0.4, 0.75, 1.5, 3, 6 µg/mL) for different durations. Then, the culture medium was changed to 10% CCK8-containing medium, and the cells were incubated for an additional 1 to 4 hours in the incubator. The absorbance was detected by a microplate reader at 450 nm.

Real-Time PCR

Total RNA was extracted using RNAiso Plus (Takara), and cDNA was generated by an RT Reagent Kit with gDNA Eraser (Takara) by the TB Green qPCR method. Real-time PCR was conducted using TB Green (Takara) according to the manufacturer's

instructions. ACTB was used as an internal control and relative mRNA expression was calculated using the $2^{-\Delta\Delta Ct}$ method. All primers are listed below:

Primers	Sense (5'-3')	Antisense (5'-3')
VEGFA	CTTGCCTTGCTGCTCTACCT	GCAGTAGCTGCGCTGATAGA
VEGFR2	GTAACCCGGAGTGACCAAGG	AACCAAGGTACTTCGCAGGG
TNF-α	GCTGCACTTTGGAGTGATCG	TCACTCGGGGTTCGAGAAGA
IL-6	TGCAATAACCACCCCTGACC	ATTTGCCGAAGAGCCCTCAG
VCAM1	GGACCACATCTACGCTGACAA	CTCCAGAGGGCCACTCAAAT
ICAM1	AGCCCAAGTTGTTGGGCATA	TGGAGTCCAGTACACGGTGA
IL-1B	CAGAAGTACCTGAGCTCGCC	AGATTCGTAGCTGGATGCCG
IL-5	TACGTGTATGCCATCCCCAC	CCCCCTTGCACAGTTTGACT
IFNG	GGCTTTTCAGCTCTGCATCG	TCTGTCACTCTCCTCTTTCCA
IL-2	ACTCAAACCTCTGGAGGAAGTG	TTCTACAATGGTTGCTGTCTCA
IL-4	CTTTGCTGCCTCCAAGAACAC	GTTCCTGTCGAGCCGTTTCA
ACTB	TTGCCGACAGGATGCAGAAGGA	AGGTGGACAGCGAGGCCAGGAT

Western Blotting

Cells were lysed in RIPA buffer supplemented with protease inhibitors and phosphatase inhibitors (MCE). The cell lysates were subjected to the BCA assay (Tiangen) for normalization. Equal amounts of protein were separated by 8% gradient SDS-PAGE gel and then transferred to polyvinylidene fluoride membranes (Millipore) for immunoblotting. The blots were incubated with 3% skim milk followed by antibody incubation. All antibodies used are listed below:

Antibody	Company	Catalog
VEGFR2	Cell Signaling Technology	#9698
ICAM1	Cell Signaling Technology	#4915

VCAM1	Cell Signaling Technology	#13362
GAPDH	Cell Signaling Technology	#5174

ELISA

Human VEGF, TNF-α and IL-6 ELISA assays were performed according to the manufacturer's instructions (Fine Biotech). Briefly, samples were diluted by 50% with dilution buffer, and then 100 μL of each sample or standard was added to each well and incubated at 37 °C for 90 min. Then, the plate contents were discarded and the plate was washed with wash buffer. After incubation with biotin-labeled antibody at 37 °C for 1 hour, HRP-streptavidin conjugate working solution was added to each well and incubated at 37 °C for 30 min. Then, TMB substrate was added to each well and incubated at 37 °C in the dark. Stop solution was added to each well when apparent gradient appeared in the standard wells, and the absorbance was read at 450 nm immediately. The standard curve was plotted, and the concentrations of the samples were calculated from the curve.

Tube Formation

Growth factor-reduced basement membrane matrix (BD) was diluted to 10 mg/mL with complete medium. Then, 50 μL of diluted matrix was added to the wells and incubated at 37 °C for one hour to obtain a solid state. hRECs (1.5×10^4) were seeded in precoated 96-well plate in a volume of 100 μL after pretreatment with 2-piperidone for 24 hours. PBS or 2-piperidone was added to the wells, and the cells were incubated at 37 °C for 6 hours. Tube formation was detected using an inverted microscope and analyzed by measuring the length of the tubes using ImageJ.

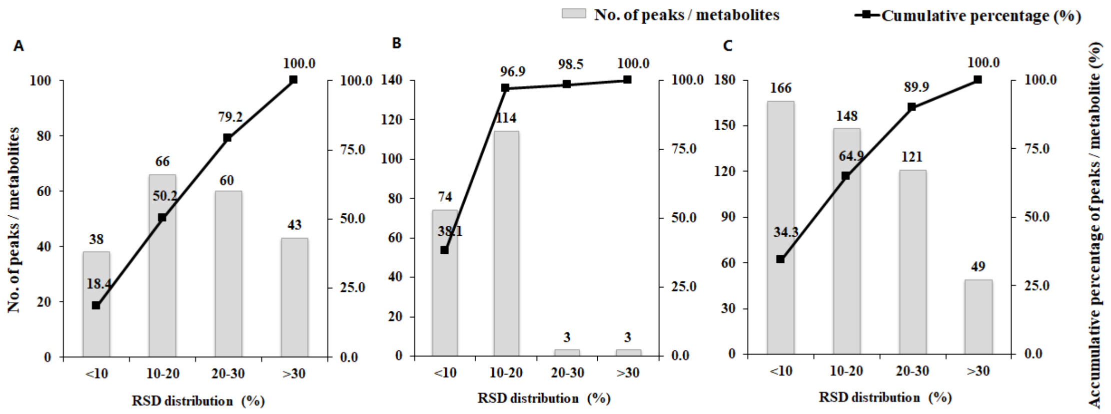

Figure S1. Stability of analytical methods based on GC-MS metabolomics (A), LC-MS metabolomics (B), and LC-MS lipidomics (C) for QCs inserted in the analysis batch after every 10 actual samples.

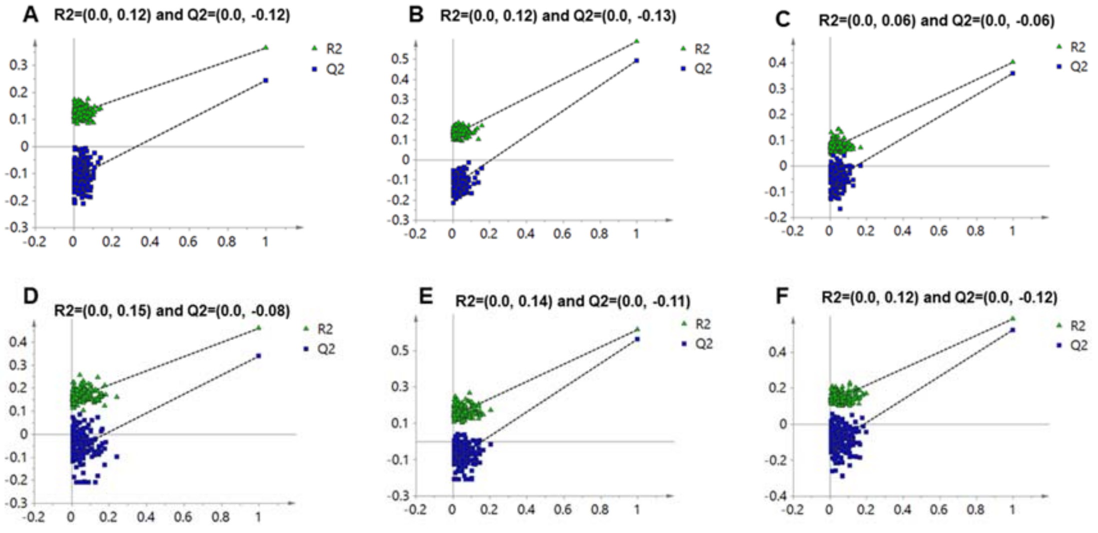

Figure S2. Cross-validation plots with a permutation test repeated 200 iterations for the PLS-DA modes of DR vs. NDR for GC-MS metabolomics (A), LC-MS metabolomics (B), and LC-MS lipidomics (C). Cross-validation plots with a permutation test repeated 200 iterations for the PLS-DA modes of NPDR vs. NDR for GC-MS metabolomics (D), LC-MS metabolomics (E), and LC-MS lipidomics (F).

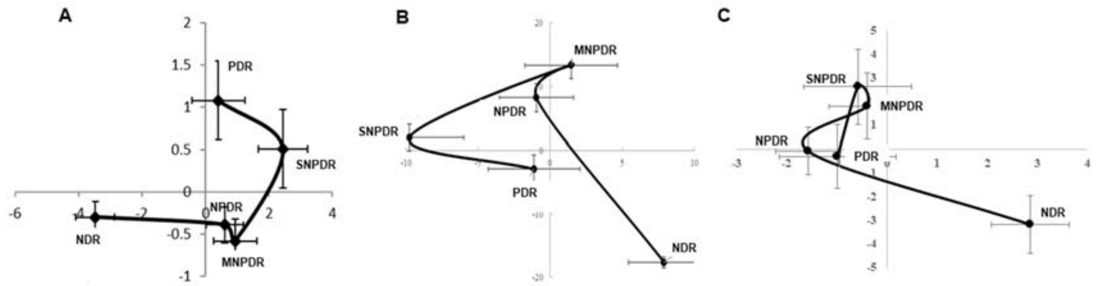

Figure S3. Molecular trajectory plots from NDR to PDR based on GC-MS metabolomics (A), LC-MS metabolomics (B). and LC-MS lipidomics (C). The trajectory plots were produced based on principal component 1 and principal component 2 of the principal component analysis model for the NDR, NPDR, MNPDR, SNPDR, PDR groups.

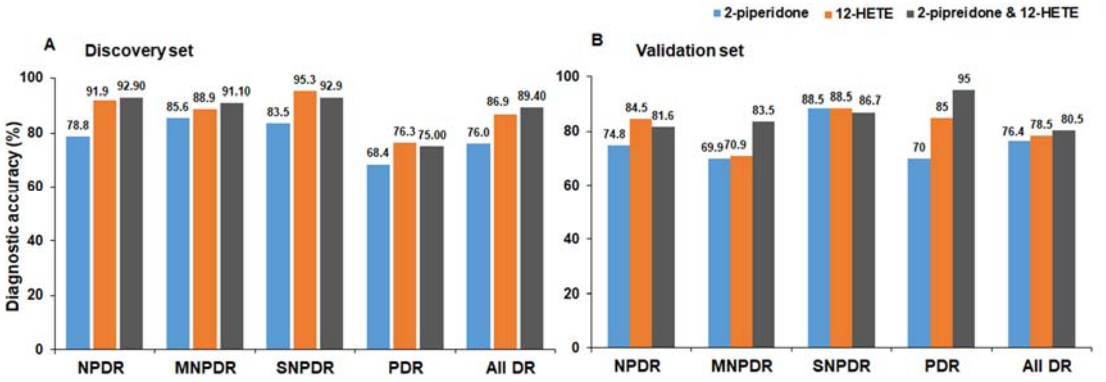

Figure S4. Diagnostic performances of 2-piperidone, 12-HETE, and both in the diagnosis of DR in the discovery and validation sets, respectively.

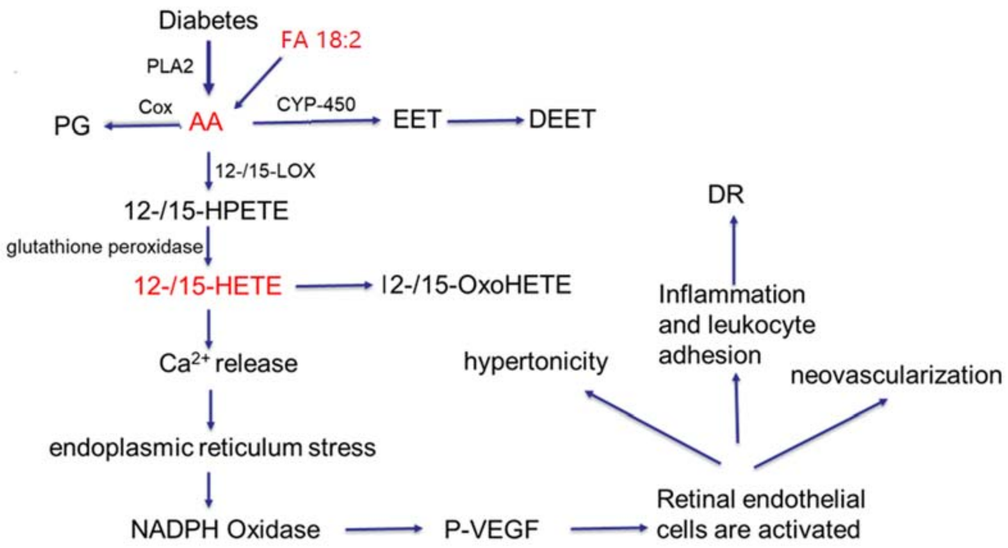

Figure S5. Cascade events involved in the pathogenesis of DR. Red text represents significant increase..

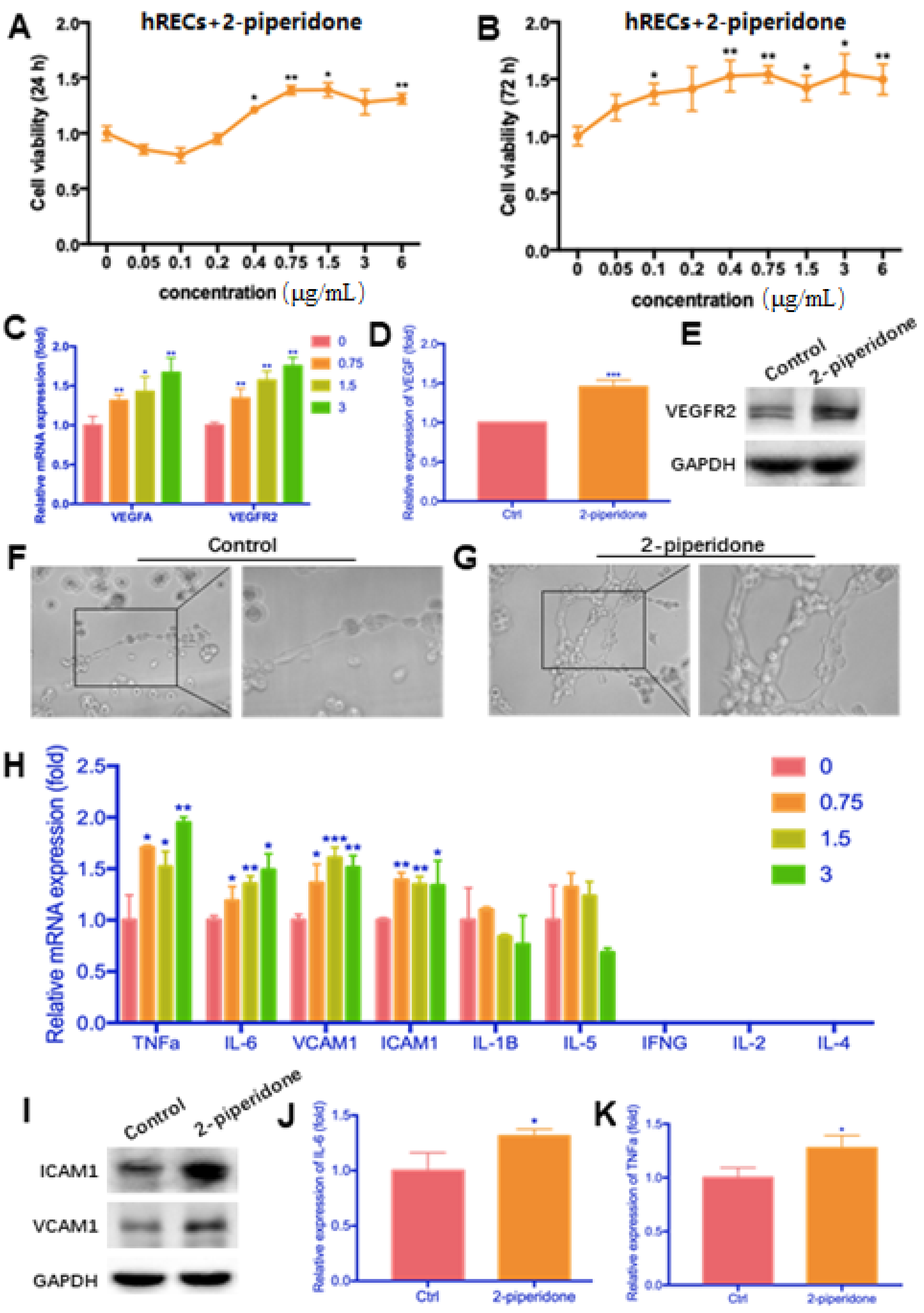

Figure S6. Functional study of the effect of 2-piperidoneon human retinal endothelial cells (hRECs). Viability plot of hRECs treated with 2-piperidone at different concentrations for 24 h (A) and 72 h (B). (C) Relative mRNA expression of VEGFA and VEGFR2 in hRECs treated with the indicated concentration of 2-piperidone. (D) Relative concentration of VEGF in culture medium of hRECs treated with vehicle or 2-piperidone. (E) Immunoblots of VEGFR2 in hRECs treated with 2-piperidone. (F-G) Representative images of tube formation in vehicle- or 2-piperidone-treated hRECs. (H) Relative mRNA expression of the indicated genes in hRECs treated with the indicated

concentration of 2-piperidone. (I) Immunoblots of ICAM1 and VCAM1 in hRECs. (J) Relative concentration of IL-6 in hRECs treated with vehicle or 2-piperidone. (K) Relative concentration of TNF-α in hRECs treated with vehicle or 2-piperidone. Data are presented as mean ± SD from at least three independent experiments, they were compared using the Student's t-test, * $p < 0.05$, ** $p < 0.01$, *** $p < 0.001$.

Table S1. No. of differential metabolites based on GC-MS metabolomics (GC-MSM), LC-MS metabolomics (LC-MSM), and LC-MS lipidomics (LC-MSL) in the discovery set. The metabolite data were compared using nonparametric tests in Wilcoxon, Mann-Whitney test and Benjamini-Hochberg-based FDR modes

Groups	No. of differential metabolites (p <0.05，FDR < 0.05)			No. of differential metabolites (p < 0.05)		
	GC-MSM	LC-MSM	LC-MSL	GC-MSM	LC-MSM	LC-MSL
NDR *vs.* NPDR	81	103	156	86	114	176
NDR *vs.* MNPDR	78	91	144	83	107	173
NDR *vs.* SNPDR	91	106	186	91	116	218
NDR *vs.* PDR	55	61	86	65	77	137
NPDR *vs.* PDR	2	11	2	13	34	30
MNPDR *vs.* PDR	4	21	5	17	40	45
SNPDR *vs.* PDR	6	0	0	17	20	18
NPDR *vs.* MNPDR	0	0	0	7	6	2
NPDR *vs.* SNPDR	8	0	0	17	26	5
MNPDR *vs.* SNPDR	0	14	0	13	32	2

Table S2. Ion pair information for targeted analysis

Metabolite	Ion Mode	Qualifier			Quantifier		
		m/z	CE	CV	m/z	CE	CV
**2-piperidone**	Positive	56.01	14	18	82.07	10	18
**Phe-d5**	Positive	179.97	26	18	125.01	14	18
**12-HETE**	Negative	257.16	14	8	179.06	14	8
**12-HETE-d8**	Negative	308.98	14	32	184.08	14	32

Table S3. The most significantly changed metabolites contributing to discriminating NPDR and DR from NDR based on the GC-MS metabolomics platform. The metabolite data were compared using nonparametric tests in Wilcoxon, Mann-Whitney test and Benjamini-Hochberg-based FDR modes Provided in a separate Excel file.

Table S4. The most significantly changed metabolites contributing to discriminating NPDR and DR from NDR based on the LC-MS metabolomics platform. The metabolite data were compared using nonparametric tests in Wilcoxon, Mann-Whitney test and Benjamini-Hochberg-based FDR modes Provided in a separate Excel file.

Table S5. The most significantly changed metabolites contributing to discriminating NPDR and DR from NDR based on the LC-MS lipidomics platform. The metabolite data were compared using nonparametric tests in Wilcoxon, Mann-Whitney test and Benjamini-Hochberg-based FDR modes Provided in a separate Excel file.

Table S3. The most significantly changed metabolites contributing to the discrimination of NPDR or DR from NDR based on the GC-MS metabolomics platform (FDR: false discovery rate).

	DR *vs.* NDR			NPDR *vs.* NDR		
**ID**	**p value**	**FDR**	**Fold change**	**p value**	**FDR**	**Fold change**
Ethylene glycol	1.33E-07	5.74E-07	1.19	2.37E-05	9.47E-05	1.15
N,N-Dimethylglycine	1.64E-02	2.26E-02	1.13	1.45E-03	3.44E-03	1.13
1-Hexanol	7.20E-02	9.15E-02	1.02	2.89E-02	4.51E-02	1.05
1,2-Propanediol	2.98E-01	3.24E-01	1.07	3.96E-01	4.61E-01	1.09
1,2-Ethenediol	4.80E-04	9.16E-04	1.17	9.61E-02	1.28E-01	1.05
Glyoxylic acid	2.00E-03	3.23E-03	1.10	4.58E-02	6.89E-02	1.08
2,3-Butanediol	4.92E-06	1.47E-05	1.70	1.01E-04	3.24E-04	1.73
2-Hydroxypyridine	2.73E-03	4.27E-03	1.05	7.75E-03	1.51E-02	1.06
Lactic acid	1.22E-12	1.42E-11	1.22	1.19E-10	2.78E-09	1.26
Glycolic acid	1.54E-01	1.79E-01	1.05	3.99E-01	4.61E-01	1.03
2-Hydroxybutyric acid	2.65E-02	3.54E-02	1.13	2.20E-02	3.57E-02	1.11
Oxalic acid	9.59E-01	9.59E-01	1.02	9.22E-01	9.33E-01	1.01
3-Hydroxypyridine	5.47E-03	8.09E-03	1.05	5.04E-02	7.38E-02	1.04
3-Hydroxybutyric acid	7.98E-02	9.91E-02	1.40	4.89E-01	5.38E-01	1.29
3-Hydroxyisobutyric acid	7.96E-04	1.44E-03	1.15	2.67E-03	5.93E-03	1.12
2-Hydroxy-3-Methylbutyric acid	9.00E-02	1.10E-01	1.15	2.55E-02	4.02E-02	1.18
4-Hydroxypyridine	1.29E-01	1.52E-01	1.03	1.51E-01	1.94E-01	1.04
Mono-Methyl phosphate	2.84E-04	5.48E-04	1.13	9.33E-04	2.46E-03	1.13
Mimosine	2.87E-01	3.16E-01	1.06	4.53E-01	5.05E-01	1.02
Hydroxyurea	1.19E-03	2.07E-03	1.17	8.38E-02	1.15E-01	1.10
Urea	6.41E-04	1.20E-03	1.13	1.49E-02	2.57E-02	1.09
Benzoic acid	3.92E-08	1.79E-07	3.20	3.57E-07	2.55E-06	2.50
Glycerol	6.57E-04	1.21E-03	1.13	2.08E-03	4.81E-03	1.19
Ethanolamine	0.00E+00	0.00E+00	1.37	7.11E-15	1.17E-12	1.40
Leucine	5.22E-08	2.31E-07	1.26	2.31E-06	1.39E-05	1.29
Phosphate	3.72E-02	4.77E-02	1.11	6.37E-02	9.17E-02	1.14
Succinic acid	8.97E-09	5.25E-08	1.18	3.37E-06	1.84E-05	1.18
Glyceric acid	3.90E-01	4.13E-01	0.97	7.16E-01	7.38E-01	0.99
2,3-Dihydroxybutanoic acid	1.40E-05	3.95E-05	1.27	4.68E-05	1.73E-04	1.25
Porphine	2.82E-01	3.12E-01	1.01	3.25E-01	3.86E-01	1.01
Fumaric acid	0.00E+00	0.00E+00	1.39	7.52E-11	2.46E-09	1.38
Nonanoic acid	2.39E-01	2.68E-01	1.03	3.76E-01	4.44E-01	1.02
2,4-Dihydroxybutanoic acid	1.17E-08	6.38E-08	1.35	7.06E-06	3.60E-05	1.27
Beta-Alanine	2.89E-13	5.93E-12	2.74	5.33E-09	7.95E-08	2.57
9-Decenoic acid	4.93E-05	1.23E-04	1.23	2.36E-04	6.90E-04	1.28
Capric acid	6.86E-03	9.95E-03	1.12	3.04E-03	6.66E-03	1.19
3-Aminoisobutyric acid	3.22E-03	4.99E-03	1.31	3.44E-03	7.23E-03	1.33
Aminomalonic acid	5.84E-05	1.41E-04	1.15	1.75E-02	2.94E-02	1.07
Malic acid	3.33E-15	9.10E-14	1.37	1.02E-10	2.78E-09	1.39
Threitol	4.01E-06	1.26E-05	1.33	1.91E-03	4.47E-03	1.14
Erythritol	5.40E-10	3.76E-09	1.35	2.36E-06	1.39E-05	1.24
Pyroglutamic acid	5.60E-07	2.09E-06	1.17	9.40E-06	4.35E-05	1.20
Erythronic acid	1.93E-06	6.60E-06	1.26	7.73E-04	2.08E-03	1.15
Threonic acid	1.30E-02	1.80E-02	0.92	1.55E-02	2.63E-02	0.89
Cysteine	4.24E-07	1.66E-06	1.22	1.79E-05	7.57E-05	1.20
Creatinine	2.90E-01	3.17E-01	1.05	4.02E-01	4.62E-01	1.02
Hypotaurine	2.00E-15	6.55E-14	1.58	1.36E-12	5.59E-11	1.58
Lyxose	9.34E-13	1.28E-11	1.42	8.82E-06	4.26E-05	1.25
Lyxosylamine	1.70E-11	1.64E-10	1.30	2.64E-06	1.50E-05	1.25
Lauric acid	1.01E-03	1.78E-03	1.18	1.09E-02	2.06E-02	1.18
Ribose	2.08E-01	2.37E-01	1.03	8.94E-01	9.11E-01	0.96
N-acetyl-L-glutamic acid	9.53E-02	1.16E-01	1.08	2.11E-01	2.60E-01	1.09
Arabitol	1.18E-10	9.69E-10	1.40	2.19E-07	1.91E-06	1.31
Rhamnose	2.01E-03	3.23E-03	1.15	4.87E-02	7.19E-02	1.11
2-Keto-D-gluconic acid	3.62E-02	4.67E-02	1.26	4.70E-01	5.21E-01	0.96
Glutamine	3.09E-02	4.05E-02	1.10	9.21E-02	1.25E-01	1.11
Phosphoethanolamine	1.91E-03	3.14E-03	1.12	1.44E-02	2.53E-02	1.10
N-Acetylornithine	4.70E-12	5.14E-11	1.49	2.66E-07	2.04E-06	1.44
Isocitric acid	1.89E-03	3.13E-03	1.15	1.30E-03	3.22E-03	1.17
Citric acid	3.97E-03	6.03E-03	1.14	2.63E-03	5.92E-03	1.18
Hypoxanthine	6.42E-12	6.58E-11	1.44	6.17E-07	4.22E-06	1.36
Glycyl-glycine	5.50E-10	3.76E-09	1.45	2.54E-05	9.93E-05	1.35
Tagatose	2.13E-04	4.30E-04	1.19	3.92E-03	8.03E-03	1.14
1,5-Anhydroglucitol	1.07E-02	1.50E-02	0.69	7.97E-01	8.17E-01	0.90
Myristic acid	1.11E-04	2.40E-04	1.19	1.92E-04	5.93E-04	1.26
N-Acetyl-L-lysine	5.21E-05	1.28E-04	1.19	3.86E-03	8.02E-03	1.17
Mannose	2.82E-04	5.48E-04	1.22	1.74E-01	2.22E-01	1.06
Lysine	3.55E-07	1.42E-06	1.22	2.17E-05	8.91E-05	1.23
Mannitol	1.12E-08	6.35E-08	1.21	4.82E-05	1.73E-04	1.18
Glucuronic acid	8.95E-01	9.06E-01	1.08	9.55E-01	9.55E-01	1.01
Isopropyl beta-D-1-Thiogalactopyranoside	2.15E-03	3.42E-03	1.15	2.25E-01	2.75E-01	1.03
3-Indoleacetic acid	1.24E-03	2.14E-03	1.25	7.49E-03	1.50E-02	1.13
Gluconic acid	2.80E-06	9.17E-06	1.44	4.80E-02	7.16E-02	1.32
Xanthine	3.55E-03	5.44E-03	1.16	1.01E-02	1.94E-02	1.13
Allo-inositol	2.14E-01	2.42E-01	1.11	7.92E-02	1.12E-01	1.18
Palmitoleic acid	7.81E-02	9.78E-02	1.22	9.34E-02	1.26E-01	1.31
Palmitic acid	1.66E-05	4.46E-05	1.17	9.44E-04	2.46E-03	1.17

Myo-Inositol	6.44E-13	1.06E-11	1.39	1.07E-09	1.95E-08	1.30
3-Indolepropionic acid	6.20E-01	6.39E-01	0.73	6.74E-01	7.04E-01	1.25
Heptadecanoic acid	4.73E-01	4.94E-01	1.06	9.47E-01	9.53E-01	1.02
Indolelactic acid	7.86E-03	1.12E-02	1.14	2.55E-02	4.02E-02	1.12
Linoleic acid	2.74E-05	7.25E-05	1.24	1.35E-03	3.27E-03	1.27
Oleic acid	5.21E-03	7.84E-03	1.17	1.30E-02	2.37E-02	1.20
Tryptophan	1.32E-06	4.74E-06	1.22	1.70E-06	1.07E-05	1.28
Stearic acid	3.03E-05	7.88E-05	1.18	2.40E-03	5.46E-03	1.17
Octanoic acid, 2-dimethylaminoethyl ester	7.30E-03	1.05E-02	1.20	3.32E-02	5.09E-02	1.21
Arachidonic acid	2.54E-04	5.01E-04	1.20	1.15E-02	2.14E-02	1.16
cis-5,8,11,14,17-Eicosapentaenoic acid	1.02E-01	1.21E-01	1.07	4.47E-01	5.03E-01	1.03
5-Methyluridine	6.19E-05	1.47E-04	1.18	3.14E-03	6.68E-03	1.13
Docosahexaenoic acid	1.02E-04	2.22E-04	1.22	4.14E-03	8.37E-03	1.16
Maltose	9.83E-10	6.45E-09	1.66	6.17E-04	1.69E-03	1.27
1-Monooleoylglycerol	9.83E-02	1.18E-01	1.07	5.23E-01	5.64E-01	1.01
Gamma-Tocopherol	2.33E-02	3.14E-02	1.14	3.30E-02	5.09E-02	1.11
Alpha-Tocopherol	5.77E-01	5.99E-01	0.99	5.22E-01	5.64E-01	0.97
Cholesterol	3.31E-01	3.57E-01	1.03	3.92E-01	4.60E-01	1.04
2-Aminobutyric acid	8.78E-05	2.00E-04	1.20	1.44E-04	4.54E-04	1.20
3-Methyl-2-oxobutanoic acid	1.11E-05	3.18E-05	1.25	9.55E-06	4.35E-05	1.36
3-Methyl-2-oxovaleric acid	6.71E-06	1.97E-05	1.27	1.41E-06	9.23E-06	1.36
Alanine	1.57E-05	4.28E-05	1.19	8.14E-05	2.78E-04	1.22
Asparagine	4.92E-07	1.88E-06	1.20	1.80E-05	7.57E-05	1.22
Aspartic acid	2.30E-08	1.11E-07	1.23	8.90E-05	2.98E-04	1.17
Citrulline	4.17E-05	1.05E-04	1.16	1.39E-02	2.48E-02	1.11
Fructose	4.89E-06	1.47E-05	1.24	1.34E-03	3.27E-03	1.18
Glucose	1.35E-01	1.59E-01	1.03	2.09E-01	2.60E-01	1.03
Glutamic acid	1.30E-09	8.21E-09	1.24	2.74E-07	2.04E-06	1.24
Glycine	6.76E-05	1.58E-04	1.12	3.09E-03	6.67E-03	1.10
Isoleucine	3.35E-11	3.05E-10	1.28	4.32E-08	4.43E-07	1.28
Methionine	3.15E-08	1.48E-07	1.28	1.89E-08	2.39E-07	1.37
Ornithine	4.44E-15	1.04E-13	1.42	1.52E-10	3.12E-09	1.36
Phenylalanine	1.72E-08	8.81E-08	1.21	7.24E-06	3.60E-05	1.19
Serine	5.73E-13	1.04E-11	1.33	4.25E-09	6.97E-08	1.28
Threonine	2.24E-10	1.75E-09	1.29	1.37E-08	1.87E-07	1.33
Trans-4-Hydroxyproline	7.88E-05	1.82E-04	1.19	9.95E-04	2.55E-03	1.13
Tyrosine	3.88E-05	9.94E-05	1.17	3.22E-05	1.23E-04	1.22
Uric acid	8.29E-02	1.02E-01	1.09	5.27E-02	7.65E-02	1.10
Uridine	1.44E-05	4.01E-05	1.24	5.04E-04	1.40E-03	1.15
Valine	1.33E-06	4.74E-06	1.18	4.96E-05	1.73E-04	1.19
Proline	3.55E-10	2.64E-09	1.34	1.08E-07	1.05E-06	1.46

Table S4. The most significantly changed metabolites contributing to the discrimination of NPDR or DR from NDR based on the LC-MS metabolomics platform (FDR: false discovery rate).

	DR *vs.* NDR			NPDR *vs.* NDR		
**ID**	**p value**	**FDR**	**Fold change**	**p value**	**FDR**	**Fold change**
12-HETE	0.00E+00	0.00E+00	5.72	0.00E+00	0.00E+00	5.57
2-Piperidone	0.00E+00	0.00E+00	3.58	0.00E+00	0.00E+00	3.51
TCA	5.10E-09	2.71E-08	2.61	2.57E-09	1.86E-08	3.66
Asp-phe	4.93E-12	3.43E-11	1.99	1.14E-06	5.68E-06	1.83
P-cresyl glucuronide	1.88E-03	4.32E-03	1.66	2.67E-01	3.61E-01	1.18
LPC 20:4 -2	0.00E+00	0.00E+00	1.66	6.66E-16	1.14E-14	1.62
LPC o-16:0	9.33E-15	8.49E-14	1.55	1.33E-10	1.16E-09	1.56
LPC O-18:0	2.38E-14	2.09E-13	1.54	6.06E-10	4.76E-09	1.52
LPC 16:1	7.32E-12	5.05E-11	1.54	1.06E-09	8.05E-09	1.57
LPC O-18:1	2.22E-16	2.30E-15	1.54	5.07E-11	4.63E-10	1.49
GCA	3.06E-05	9.66E-05	1.53	9.69E-05	3.43E-04	2.28
Phenylacetylglutamine	1.55E-05	5.19E-05	1.51	3.77E-02	7.09E-02	1.13
GCDCA-3-glucu	5.85E-03	1.19E-02	1.48	1.47E-02	3.14E-02	1.51
LPC O-16:1	0.00E+00	0.00E+00	1.47	3.61E-13	4.34E-12	1.51
LPC 14:0	1.17E-07	5.26E-07	1.46	1.93E-07	1.10E-06	1.52
LPC 20:2	1.98E-13	1.60E-12	1.43	4.87E-10	3.91E-09	1.43
LPC 16:0	0.00E+00	0.00E+00	1.41	1.22E-14	1.78E-13	1.42
LPC 22:4	1.51E-07	6.68E-07	1.40	4.24E-05	1.60E-04	1.34
LPE 20:1	3.06E-11	1.97E-10	1.40	1.08E-08	7.20E-08	1.44
γ-Glu-Leu	4.39E-11	2.77E-10	1.37	1.50E-08	9.90E-08	1.38
LPC 18:0	6.66E-16	6.70E-15	1.37	7.94E-12	7.96E-11	1.37
Choline	0.00E+00	0.00E+00	1.36	8.22E-15	1.23E-13	1.36
Phe-Phe	7.78E-05	2.33E-04	1.34	1.78E-03	4.81E-03	1.28
Sphingosine-1-phosphate	5.44E-09	2.88E-08	1.33	2.73E-06	1.28E-05	1.29
LPC 16:0	0.00E+00	0.00E+00	1.33	2.62E-12	2.86E-11	1.34
LPC 15:0	1.63E-07	7.19E-07	1.32	9.91E-06	4.17E-05	1.33
carnitine C18:2	7.14E-07	2.90E-06	1.32	2.29E-05	9.05E-05	1.35
3-(1-Pyrazolyl)-L-alanine	0.00E+00	0.00E+00	1.31	0.00E+00	0.00E+00	1.34
LPC 20:4 -1	1.50E-07	6.67E-07	1.30	1.28E-04	4.40E-04	1.25
UDCA	1.80E-01	2.43E-01	1.30	2.08E-01	2.93E-01	1.17
Hypoxanthine	2.50E-08	1.22E-07	1.30	7.37E-05	2.65E-04	1.25
Carnitine C5:0	8.24E-07	3.31E-06	1.29	4.29E-05	1.62E-04	1.26
FA 20:4	1.63E-08	8.15E-08	1.28	6.71E-07	3.45E-06	1.30
3,4,5-TRIMETHOXYCINNA	2.04E-03	4.66E-03	1.28	1.67E-03	4.54E-03	1.33
LPE 16:1	2.31E-01	3.01E-01	1.28	1.46E-01	2.19E-01	1.22
LPE 18:0	7.84E-09	4.08E-08	1.28	3.96E-05	1.50E-04	1.23
D-Citramalic Acid	3.48E-11	2.21E-10	1.27	1.03E-05	4.34E-05	1.19
Hippuric acid	4.19E-02	6.76E-02	1.25	3.49E-01	4.48E-01	1.07
LPC 17:0	3.93E-06	1.45E-05	1.25	3.18E-04	1.01E-03	1.22
LPC 18:1	4.25E-08	2.01E-07	1.24	2.92E-06	1.36E-05	1.25
FA 16:2	1.83E-04	5.12E-04	1.24	2.17E-05	8.65E-05	1.29
LPC 20:0	3.38E-05	1.06E-04	1.24	2.77E-05	1.08E-04	1.28
N-acetylaspartate	4.75E-04	1.24E-03	1.24	3.66E-03	9.11E-03	1.20
GUDCS	2.45E-01	3.16E-01	1.23	2.12E-01	2.98E-01	1.22
LPC 22:6	3.80E-05	1.18E-04	1.22	3.53E-03	8.83E-03	1.15
Uridine	6.27E-12	4.34E-11	1.21	6.91E-09	4.69E-08	1.21
GCDCS/GDCS	1.72E-02	3.07E-02	1.21	3.79E-02	7.13E-02	1.24
Hydrocortisone	1.49E-03	3.52E-03	1.20	2.34E-03	6.15E-03	1.24
Glutamic acid	2.11E-08	1.04E-07	1.20	1.43E-05	5.85E-05	1.19
PE o-38:7	5.59E-04	1.44E-03	1.20	5.73E-02	1.01E-01	1.18
LPE 16:0	1.43E-04	4.10E-04	1.19	2.14E-02	4.35E-02	1.13
FA 16:1	1.51E-02	2.75E-02	1.19	1.25E-02	2.70E-02	1.27
FA 16:0	5.71E-04	1.47E-03	1.19	1.66E-03	4.51E-03	1.20
L-Tryptophan	1.72E-05	5.71E-05	1.18	6.57E-05	2.39E-04	1.23
Creatinine	6.05E-04	1.55E-03	1.18	1.14E-02	2.49E-02	1.11
SM 36:3	2.69E-04	7.35E-04	1.18	2.57E-02	5.10E-02	1.12
Abietic acid	2.69E-02	4.56E-02	1.18	4.07E-02	7.59E-02	1.16
Proline	2.30E-07	9.95E-07	1.18	6.34E-06	2.78E-05	1.26
FA 14:0	3.58E-03	7.71E-03	1.18	1.56E-03	4.26E-03	1.24
LPC 20:5	1.51E-02	2.75E-02	1.17	6.20E-02	1.08E-01	1.16
LPC 20:3	1.32E-03	3.13E-03	1.16	6.58E-03	1.54E-02	1.16
Biliverdin	5.75E-01	6.52E-01	1.16	8.20E-02	1.36E-01	1.30
Phenyl sulfate	2.55E-02	4.35E-02	1.15	3.94E-01	4.92E-01	1.02
Tyrosine	3.22E-05	1.02E-04	1.15	1.20E-05	4.99E-05	1.20
Indolelactic acid	7.70E-03	1.51E-02	1.15	3.54E-02	6.73E-02	1.13
FA 20:3	1.25E-02	2.33E-02	1.15	4.51E-03	1.09E-02	1.19
FA 16:3	2.84E-02	4.78E-02	1.15	3.58E-03	8.94E-03	1.19
FA 14:1	9.59E-02	1.40E-01	1.14	4.05E-02	7.55E-02	1.22
Carnitine C4:0	3.80E-02	6.21E-02	1.14	1.94E-01	2.77E-01	1.08
Carnitine C18:0	3.19E-02	5.31E-02	1.14	9.08E-02	1.48E-01	1.11
Farnesylacetone	3.75E-03	8.06E-03	1.14	1.05E-02	2.31E-02	1.15

Homoserine	8.24E-07	3.31E-06	1.14	6.40E-07	3.32E-06	1.17
LPC 20:1	2.17E-03	4.93E-03	1.14	1.11E-02	2.43E-02	1.14
FA 18:2	4.09E-03	8.68E-03	1.13	1.08E-02	2.38E-02	1.14
Hydroxycaproic acid	3.59E-06	1.33E-05	1.13	1.24E-05	5.14E-05	1.13
SM 34:2	1.44E-03	3.40E-03	1.13	3.75E-02	7.06E-02	1.10
Decadienoylcarnitine	3.79E-01	4.61E-01	1.13	2.62E-01	3.55E-01	1.18
LPE 18:2 -2	1.70E-02	3.04E-02	1.13	2.33E-02	4.67E-02	1.12
Valine	2.19E-05	7.12E-05	1.13	7.14E-04	2.09E-03	1.13
LPC 18:2	2.89E-03	6.37E-03	1.13	8.34E-03	1.90E-02	1.13
β-Nicotyrine	8.24E-04	2.06E-03	1.13	2.79E-04	8.99E-04	1.18
Leucine/Isoleucine	8.48E-07	3.40E-06	1.13	7.95E-05	2.85E-04	1.13
Methylisoquinoline	1.03E-03	2.52E-03	1.13	3.24E-04	1.03E-03	1.18
Ornithine	1.13E-06	4.46E-06	1.13	9.97E-05	3.51E-04	1.13
LPC 18:3	1.56E-01	2.14E-01	1.12	3.83E-02	7.20E-02	1.16
Hydroxyquinoline	1.08E-03	2.62E-03	1.12	3.02E-04	9.64E-04	1.18
SM 33:1	5.86E-02	9.15E-02	1.12	3.95E-01	4.93E-01	1.05
FA 18:0	7.25E-03	1.44E-02	1.12	1.03E-02	2.29E-02	1.12
Tryptophan	1.49E-03	3.51E-03	1.12	5.02E-04	1.53E-03	1.17
FA 22:6	8.11E-02	1.21E-01	1.12	2.96E-02	5.74E-02	1.16
LPC 22:5	3.44E-03	7.44E-03	1.11	3.22E-02	6.19E-02	1.07
SM 32:1	3.28E-02	5.44E-02	1.11	1.07E-01	1.70E-01	1.10
Carnitine C8:1	6.45E-01	7.14E-01	1.11	2.16E-01	3.02E-01	1.19
FA 18:4	5.40E-01	6.19E-01	1.11	2.15E-01	3.00E-01	1.15
Asparagine	5.76E-03	1.17E-02	1.11	1.99E-01	2.83E-01	1.03
FA 20:0	7.13E-02	1.08E-01	1.10	5.77E-03	1.37E-02	1.16
Indoline	1.21E-03	2.91E-03	1.10	1.36E-02	2.93E-02	1.09
FA 15:0	6.20E-02	9.60E-02	1.10	2.89E-02	5.63E-02	1.13
LPE 18:1	6.43E-02	9.91E-02	1.10	9.98E-02	1.60E-01	1.07
Methionine	4.86E-04	1.27E-03	1.10	1.38E-04	4.73E-04	1.15
Phenylalanine	1.04E-03	2.54E-03	1.10	9.95E-03	2.22E-02	1.09
Myoinositol	6.22E-03	1.26E-02	1.09	3.10E-01	4.06E-01	1.02
1,7-Dimethylxanthine	1.24E-01	1.76E-01	1.09	5.35E-01	6.26E-01	1.22
Carnitine C5:1	6.80E-02	1.04E-01	1.08	1.88E-01	2.70E-01	1.02
FA 24:0	9.65E-01	9.73E-01	1.08	3.56E-01	4.55E-01	1.08
Palmitoleic acid	1.06E-01	1.53E-01	1.08	6.62E-02	1.14E-01	1.14
Mannitol	9.98E-04	2.45E-03	1.08	2.22E-05	8.81E-05	1.14
Carnitine C10:1	2.03E-01	2.69E-01	1.08	4.62E-01	5.59E-01	1.07
Carnitine C16:0	2.74E-01	3.48E-01	1.08	1.73E-01	2.52E-01	1.09
Mannose	5.93E-03	1.20E-02	1.08	2.90E-01	3.85E-01	1.02
1,3-Dimethyluric acid	6.46E-03	1.30E-02	1.08	3.23E-01	4.20E-01	1.02
FA 20:3	1.46E-01	2.03E-01	1.08	7.10E-02	1.20E-01	1.10
Phenylacetamide	2.01E-03	4.61E-03	1.07	7.73E-05	2.77E-04	1.12
Lysine	2.46E-04	6.76E-04	1.07	8.02E-04	2.32E-03	1.08
FA 10:0	6.41E-01	7.11E-01	1.07	2.96E-01	3.92E-01	1.13
FA 18:3	4.77E-01	5.59E-01	1.07	3.88E-01	4.86E-01	1.06
FA 17:0	3.22E-01	4.00E-01	1.07	3.22E-01	4.19E-01	1.06
Pyridoxamine	1.88E-03	4.33E-03	1.06	2.04E-03	5.43E-03	1.08
SM 32:2	2.23E-01	2.93E-01	1.06	2.17E-01	3.03E-01	1.07
FA 18:1	2.04E-01	2.71E-01	1.05	3.21E-01	4.18E-01	1.04
Citric acid	2.70E-01	3.44E-01	1.05	2.40E-01	3.30E-01	1.07
SM 34:1	8.98E-02	1.33E-01	1.05	7.56E-01	8.18E-01	1.01
Pro-Leu	9.41E-01	9.55E-01	1.04	6.03E-01	6.88E-01	0.96
LPE 18:2 -1	6.93E-01	7.56E-01	1.04	5.56E-01	6.46E-01	1.02
CDCA	1.54E-01	2.12E-01	1.04	5.88E-02	1.03E-01	1.08
Uric acid	2.23E-01	2.92E-01	1.04	1.97E-01	2.81E-01	1.04
13-cis-acitretin	5.62E-05	1.71E-04	1.03	2.03E-04	6.76E-04	1.03
Carnitine C14:2	7.70E-01	8.21E-01	1.03	7.03E-01	7.71E-01	1.05
Carnitine C18:1	8.72E-01	9.02E-01	1.03	7.35E-01	8.00E-01	1.02
FA 24:4	2.25E-01	2.94E-01	1.03	1.23E-01	1.90E-01	1.04
LPE 22:6	7.67E-01	8.18E-01	1.02	5.63E-01	6.52E-01	0.95
Glutamine	6.42E-01	7.12E-01	1.02	6.27E-01	7.09E-01	1.03
SM 34:0	8.35E-01	8.73E-01	1.02	2.25E-01	3.13E-01	0.97
LPE 20:4	8.00E-01	8.46E-01	1.02	5.81E-01	6.68E-01	0.97
SM 36:2	9.45E-01	9.58E-01	1.02	9.93E-02	1.59E-01	0.92
LPE 22:5	6.84E-01	7.49E-01	1.01	7.72E-01	8.31E-01	0.98
FA 18:3	8.70E-01	9.01E-01	1.01	5.10E-01	6.05E-01	1.02
PE o-38:6	5.95E-01	6.69E-01	1.01	6.50E-01	7.29E-01	0.96
Carnitine C3:0	4.14E-01	4.96E-01	1.00	5.43E-01	6.33E-01	0.99
TUDCA	1.04E-02	1.97E-02	1.00	1.80E-01	2.60E-01	1.25
GDCA/GCDCA	1.71E-01	2.32E-01	1.00	2.84E-01	3.79E-01	1.24
Carnitine C16:2	8.73E-01	9.03E-01	0.99	7.53E-01	8.16E-01	1.01
Arginine	4.11E-01	4.92E-01	0.99	4.13E-01	5.11E-01	0.98
Carnitine C16:1	5.05E-01	5.84E-01	0.99	9.25E-01	9.47E-01	0.99
2,6-Dichlorobenzoic Acid	1.94E-02	3.41E-02	0.98	4.21E-01	5.20E-01	0.99
Carnitine C8:0	9.87E-01	9.90E-01	0.98	5.37E-01	6.28E-01	0.96
Carnitine C14:0	2.48E-01	3.20E-01	0.97	7.80E-01	8.38E-01	0.98
LPE 20:3	7.11E-01	7.71E-01	0.97	6.32E-01	7.13E-01	0.94
Monoethylhexyl phthalate	6.10E-06	2.17E-05	0.97	1.69E-04	5.70E-04	0.97

PE o-36:5	7.68E-01	8.20E-01	0.97	6.53E-01	7.32E-01	0.93
PE 34:2	7.63E-02	1.15E-01	0.97	1.93E-02	3.98E-02	0.90
GUDCA	4.83E-01	5.64E-01	0.96	2.96E-01	3.92E-01	1.01
Homocysteine	1.09E-01	1.58E-01	0.96	2.27E-02	4.57E-02	0.91
FA 19:0	4.07E-01	4.88E-01	0.96	9.52E-01	9.66E-01	0.98
PC 36:4	8.88E-03	1.72E-02	0.95	4.48E-03	1.09E-02	0.93
LCA	5.04E-05	1.54E-04	0.93	2.39E-06	1.13E-05	0.89
Carnitine C12:0	9.93E-02	1.45E-01	0.92	1.88E-01	2.70E-01	0.94
SM 36:1	3.87E-03	8.27E-03	0.92	1.19E-04	4.13E-04	0.86
Carnitine C10:0	1.55E-01	2.13E-01	0.92	9.43E-02	1.53E-01	0.91
Carnitine C12:1	1.02E-01	1.49E-01	0.92	1.67E-01	2.45E-01	0.91
FA 11:1	1.58E-02	2.85E-02	0.91	2.10E-01	2.95E-01	0.97
Anabasine	2.19E-06	8.39E-06	0.91	1.34E-03	3.69E-03	0.92
PC 38:4	4.47E-05	1.38E-04	0.91	1.12E-05	4.70E-05	0.87
p-xylene	5.37E-09	2.85E-08	0.91	5.28E-06	2.35E-05	0.91
PC 34:2	4.73E-10	2.75E-09	0.91	9.91E-06	4.17E-05	0.91
Carnitine C14:1	1.08E-01	1.57E-01	0.90	3.47E-01	4.46E-01	0.91
Caffeine	6.30E-01	7.00E-01	0.90	8.83E-01	9.17E-01	1.14
Carnitine C2:0	2.66E-03	5.92E-03	0.90	3.90E-03	9.66E-03	0.87
PC 38:5	1.41E-06	5.51E-06	0.89	3.66E-07	1.99E-06	0.86
Carnitine	7.79E-06	2.73E-05	0.89	1.28E-03	3.55E-03	0.90
PC 34:4	9.71E-02	1.42E-01	0.88	1.55E-01	2.30E-01	0.85
CA	1.14E-01	1.63E-01	0.86	2.06E-01	2.91E-01	0.97
Fatty acyl amide C16:0	1.39E-04	4.00E-04	0.84	9.64E-04	2.74E-03	0.82
Hexadecan-1-ol	5.52E-03	1.13E-02	0.84	4.41E-03	1.07E-02	0.79
Epinephrine	2.49E-08	1.22E-07	0.84	2.04E-06	9.78E-06	0.82
PC 32:2	1.09E-03	2.65E-03	0.84	2.30E-02	4.62E-02	0.83
PC o-38:5	7.84E-08	3.58E-07	0.83	1.24E-09	9.28E-09	0.77
PC 36:5	1.37E-05	4.66E-05	0.83	2.73E-04	8.83E-04	0.81
PC 34:1	2.22E-16	2.30E-15	0.82	8.87E-12	8.82E-11	0.80
Indolepropionic acid	9.44E-01	9.57E-01	0.81	5.80E-01	6.66E-01	1.30
PC 34:3	4.32E-09	2.30E-08	0.80	5.88E-06	2.59E-05	0.80
Serotonin	2.57E-01	3.30E-01	0.79	5.47E-01	6.37E-01	0.89
PC o-34:1	2.07E-14	1.83E-13	0.77	3.51E-12	3.76E-11	0.74
5-Formylsalicylic acid	9.61E-03	1.84E-02	0.76	2.09E-01	2.94E-01	0.91
PC 32:0	4.22E-15	3.95E-14	0.76	5.16E-12	5.37E-11	0.72
5-Methoxysalicylic acid	9.45E-02	1.39E-01	0.74	8.92E-01	9.23E-01	0.87
PC 30:0	4.51E-06	1.64E-05	0.73	6.69E-04	1.97E-03	0.74
PC 33:2	3.55E-15	3.36E-14	0.72	1.02E-11	1.01E-10	0.69
PC 32:1	1.12E-09	6.29E-09	0.72	1.33E-05	5.49E-05	0.76
PC 33:1	0.00E+00	0.00E+00	0.68	5.39E-11	4.91E-10	0.67
Amlnocydohexanecarboxyli	4.52E-08	2.13E-07	0.48	1.44E-06	7.02E-06	0.46

Table S5. The most significantly changed metabolites contributing to the discrimination of NPDR or DR from NDR based on the LC-MS lipidomics platform (FDR: false discovery rate).

	DR *vs.* NDR			NPDR *vs.* NDR		
**ID**	**p value**	**false discovery rate (FDR)**	**Fold change**	**p value**	**false discovery rate (FDR)**	**Fold change**
Cer(d16:1/24:1)	1.08E-04	4.58E-04	1.19	3.11E-03	1.25E-02	1.17
Cer(d18:1/16:0)	3.45E-04	1.30E-03	1.16	4.29E-03	1.62E-02	1.13
Cer(d18:1/18:0)	4.78E-03	1.32E-02	1.24	2.98E-01	5.17E-01	1.09
Cer(d18:1/20:0)	1.39E-01	2.31E-01	1.09	9.12E-01	9.54E-01	0.99
Cer(d18:1/22:0)	9.21E-06	4.97E-05	1.21	1.93E-04	1.10E-03	1.21
Cer(d18:1/24:0)	1.40E-07	1.35E-06	1.33	4.37E-05	3.31E-04	1.32
Cer(d18:1/24:1)	2.25E-01	3.47E-01	0.90	7.21E-02	1.61E-01	0.85
Cer(d18:2/22:0),Cer(d16:1/24:1)	1.02E-05	5.37E-05	1.26	1.34E-03	5.98E-03	1.24
Cer(d18:2/24:1)	2.47E-01	3.77E-01	1.03	9.56E-01	9.83E-01	0.97
CerG1(d18:1/16:0)	3.12E-03	9.49E-03	1.17	2.53E-02	6.65E-02	1.13
CerG1(d18:1/22:0)	2.46E-04	9.93E-04	1.17	2.55E-04	1.40E-03	1.19
CerG1(d18:1/24:0)	1.12E-05	5.74E-05	1.30	2.74E-04	1.48E-03	1.29
CerG2(d16:1/16:1)	5.11E-05	2.37E-04	1.23	1.49E-03	6.56E-03	1.21
CerG2(d18:1/16:0)	2.00E-03	6.31E-03	1.14	1.12E-02	3.37E-02	1.14
CerG2(d18:1/24:1)	2.71E-01	4.07E-01	1.07	4.88E-01	7.05E-01	1.05
CerG2(d18:2/16:0)	2.02E-01	3.14E-01	1.07	3.74E-01	6.01E-01	1.05
ChE (15:0)	4.81E-01	6.19E-01	0.98	4.65E-01	6.82E-01	0.95
ChE (16:0)	3.31E-01	4.76E-01	1.03	3.78E-01	6.05E-01	1.02
ChE (17:0)	9.56E-02	1.69E-01	1.11	7.36E-02	1.63E-01	1.08
ChE (18:3)	4.11E-01	5.61E-01	1.10	2.68E-01	4.79E-01	1.12
ChE(18:1)	2.05E-03	6.41E-03	1.16	1.08E-02	3.31E-02	1.15
ChE(18:2)	1.99E-03	6.31E-03	1.15	8.34E-03	2.71E-02	1.15
ChE(20:3)	7.93E-03	1.98E-02	1.16	1.35E-02	3.90E-02	1.13
ChE(20:4)	2.89E-05	1.37E-04	1.27	2.06E-03	8.73E-03	1.21
ChE(20:5)	1.84E-02	4.15E-02	1.19	1.21E-01	2.56E-01	1.09
ChE(22:6)	2.63E-05	1.28E-04	1.28	1.03E-03	4.79E-03	1.21
DG(16:0/18:1)	2.87E-04	1.11E-03	1.19	5.81E-03	2.01E-02	1.08
DG(16:0/18:2),DG(16:1_18:1)	8.99E-07	6.47E-06	1.37	1.07E-04	6.54E-04	1.27
DG(18:1/18:1)	3.51E-03	1.04E-02	1.14	3.18E-02	8.19E-02	1.01
DG(18:1/18:2)	9.77E-05	4.22E-04	1.22	5.46E-03	1.92E-02	1.08
DG(18:1/20:3)	4.01E-03	1.16E-02	1.26	4.72E-02	1.16E-01	1.13
DG(18:1/20:4)	6.92E-03	1.80E-02	1.23	6.07E-02	1.41E-01	1.06
DG(18:1/22:6),DG(18:2_22:5)	5.87E-03	1.56E-02	1.29	6.86E-02	1.55E-01	1.04
DG(18:2/18:2),DG(18:1/18:3)	8.03E-06	4.50E-05	1.36	1.07E-03	4.94E-03	1.21
DG(18:2/20:4)	6.79E-05	3.02E-04	1.39	4.02E-03	1.54E-02	1.19
DG(18:2/22:6)	2.71E-04	1.06E-03	1.47	9.16E-03	2.91E-02	1.19
DG(18:3/18:2)	4.96E-03	1.36E-02	1.37	5.70E-02	1.35E-01	1.16
DG(20:3/18:2)	9.41E-03	2.28E-02	1.23	6.72E-02	1.54E-01	1.05
DG(20:5/18:2)	1.11E-01	1.92E-01	1.34	2.92E-01	5.12E-01	1.10
FA(14:0)	1.21E-03	4.02E-03	1.17	8.02E-04	3.89E-03	1.24
FA(14:1)	1.08E-02	2.55E-02	1.22	2.60E-03	1.07E-02	1.30
FA(16:0)	2.05E-06	1.36E-05	1.22	4.87E-05	3.63E-04	1.25
FA(16:1)	3.27E-03	9.81E-03	1.22	2.85E-03	1.16E-02	1.30
FA(17:0)	3.18E-04	1.21E-03	1.23	1.25E-03	5.69E-03	1.25
FA(17:1)	3.18E-03	9.62E-03	1.20	9.41E-03	2.97E-02	1.24
FA(17:2)	2.18E-05	1.07E-04	1.29	1.06E-04	6.54E-04	1.34
FA(18:1)	2.75E-04	1.07E-03	1.21	1.61E-03	7.02E-03	1.24
FA(18:2)	2.81E-07	2.33E-06	1.30	8.83E-06	9.08E-05	1.34
FA(18:3)	2.52E-01	3.84E-01	1.08	1.71E-01	3.44E-01	1.11
FA(18:4)	1.61E-01	2.60E-01	1.15	4.28E-02	1.07E-01	1.22
FA(20:1)	9.13E-01	9.43E-01	1.04	7.74E-01	8.93E-01	1.08
FA(20:2)	4.31E-06	2.58E-05	1.33	7.23E-05	4.96E-04	1.39
FA(20:3)	2.16E-04	8.87E-04	1.23	1.01E-04	6.30E-04	1.31
FA(20:4)	1.30E-12	2.16E-11	1.48	2.18E-10	4.09E-09	1.54
FA(20:5)	5.04E-03	1.37E-02	1.27	6.06E-03	2.06E-02	1.31
FA(22:2)	1.89E-01	2.99E-01	1.13	7.28E-02	1.62E-01	1.20
FA(22:3)	2.07E-02	4.58E-02	1.20	1.25E-02	3.66E-02	1.27
FA(22:4)	1.26E-03	4.14E-03	1.25	3.00E-03	1.21E-02	1.29
FA(22:5)	7.69E-03	1.94E-02	1.18	3.25E-03	1.29E-02	1.25
FA(22:6)	1.33E-03	4.33E-03	1.24	3.82E-04	1.96E-03	1.32
FA(24:2)	1.32E-04	5.54E-04	1.28	8.92E-04	4.19E-03	1.30
FA(24:3)	1.77E-01	2.84E-01	1.11	2.24E-01	4.27E-01	1.08
FA(24:4)	5.75E-05	2.64E-04	1.31	4.09E-04	2.08E-03	1.36
FA(24:5)	8.03E-03	1.99E-02	1.15	2.33E-03	9.76E-03	1.22
FA(24:6)	1.89E-01	2.99E-01	1.11	4.47E-02	1.11E-01	1.20
FA(26:1)	1.15E-03	3.85E-03	0.81	1.07E-02	3.29E-02	0.80
LPC(14:0)	7.77E-15	1.53E-13	1.74	8.04E-13	1.93E-11	1.86
LPC(15:0)	0.00E+00	0.00E+00	1.62	4.22E-15	1.40E-13	1.61
LPC(15:1)	3.11E-07	2.44E-06	1.28	1.74E-05	1.61E-04	1.27
LPC(16:0)	0.00E+00	0.00E+00	1.70	0.00E+00	0.00E+00	1.73
LPC(16:1)	0.00E+00	0.00E+00	1.69	0.00E+00	0.00E+00	1.75

LPC(17:0)	1.87E-14	3.36E-13	1.44	5.39E-10	8.96E-09	1.44
LPC(17:1)	0.00E+00	0.00E+00	1.45	6.37E-13	1.84E-11	1.48
LPC(18:0)	0.00E+00	0.00E+00	1.80	0.00E+00	0.00E+00	1.85
LPC(18:1)	0.00E+00	0.00E+00	1.44	7.28E-13	1.85E-11	1.45
LPC(18:2)	5.05E-09	5.90E-08	1.31	2.58E-07	3.38E-06	1.34
LPC(18:3)	0.00E+00	0.00E+00	1.43	4.44E-16	1.74E-14	1.43
LPC(18:4)	0.00E+00	0.00E+00	1.65	0.00E+00	0.00E+00	1.66
LPC(20:0)	2.16E-11	3.21E-10	1.38	3.92E-10	7.05E-09	1.44
LPC(20:1)	1.11E-06	7.71E-06	1.23	3.19E-05	2.55E-04	1.24
LPC(20:3)	0.00E+00	0.00E+00	1.72	4.44E-16	1.74E-14	1.74
LPC(20:4)	3.55E-15	7.67E-14	1.37	1.55E-10	3.04E-09	1.37
LPC(20:5)	2.05E-07	1.85E-06	1.25	1.87E-05	1.68E-04	1.25
LPC(22:0)	5.70E-04	2.06E-03	1.18	1.93E-04	1.10E-03	1.26
LPC(22:1)	7.39E-01	8.15E-01	0.72	5.89E-01	7.76E-01	0.80
LPC(22:3)	7.97E-11	1.15E-09	1.47	8.93E-09	1.38E-07	1.53
LPC(22:4)	2.38E-07	2.02E-06	1.33	1.67E-05	1.60E-04	1.35
LPC(22:6)	1.71E-14	3.21E-13	1.45	1.35E-10	2.78E-09	1.42
LPC(24:0)	1.80E-05	8.96E-05	1.21	3.92E-05	3.03E-04	1.27
LPC(24:1)	3.89E-01	5.37E-01	0.88	2.35E-01	4.42E-01	0.85
LPC-O 16:1	0.00E+00	0.00E+00	1.67	0.00E+00	0.00E+00	1.71
LPC-O 18:0	0.00E+00	0.00E+00	1.62	1.11E-15	4.00E-14	1.63
LPC-O 18:1	0.00E+00	0.00E+00	1.75	0.00E+00	0.00E+00	1.77
LPC-O 18:3	0.00E+00	0.00E+00	1.39	6.93E-13	1.85E-11	1.38
LPC-O 20:0	7.70E-01	8.36E-01	1.01	1.35E-01	2.78E-01	1.06
LPC-O 20:1	1.07E-09	1.36E-08	1.36	9.74E-07	1.20E-05	1.36
LPE(16:0)	7.55E-15	1.53E-13	1.43	5.32E-10	8.96E-09	1.41
LPE(16:0p)	0.00E+00	0.00E+00	1.99	0.00E+00	0.00E+00	2.07
LPE(18:0)	0.00E+00	0.00E+00	1.60	4.95E-14	1.53E-12	1.62
LPE(18:0p)	0.00E+00	0.00E+00	2.02	0.00E+00	0.00E+00	2.06
LPE(18:1)	1.66E-07	1.56E-06	1.35	2.70E-06	3.16E-05	1.37
LPE(18:1p)	0.00E+00	0.00E+00	1.80	0.00E+00	0.00E+00	1.86
LPE(18:2)	4.56E-03	1.28E-02	1.16	9.79E-03	3.06E-02	1.17
LPE(20:4)	3.06E-04	1.17E-03	1.25	4.77E-03	1.75E-02	1.24
LPE(22:5)	3.69E-03	1.08E-02	1.24	4.87E-03	1.75E-02	1.27
LPE(22:6)	6.11E-04	2.18E-03	1.24	1.29E-02	3.75E-02	1.19
OAHFA(16:0/18:2)	1.20E-02	2.79E-02	1.10	1.76E-01	3.51E-01	1.08
OAHFA(18:0/18:2),OAHFA(18:1_18:1)	2.56E-06	1.63E-05	1.38	3.53E-04	1.84E-03	1.40
OAHFA(18:1/18:0)	1.08E-03	3.65E-03	1.29	1.80E-02	4.96E-02	1.31
OAHFA(18:1/20:3)	4.70E-04	1.74E-03	1.19	1.09E-02	3.33E-02	1.19
OAHFA(18:2/18:1)	5.33E-07	4.11E-06	1.44	5.80E-05	4.17E-04	1.47
OAHFA(18:2/22:6)	3.05E-09	3.76E-08	1.32	3.80E-07	4.83E-06	1.35
PC 38:7	7.02E-03	1.82E-02	1.12	1.89E-02	5.11E-02	1.13
PC(14:0/18:2)	7.16E-01	7.99E-01	0.97	5.22E-01	7.27E-01	1.02
PC(14:0/22:4)	7.10E-05	3.10E-04	1.19	9.10E-03	2.91E-02	1.14
PC(15:0/18:2)	6.51E-01	7.48E-01	0.97	7.35E-01	8.68E-01	0.97
PC(15:0/20:4)	1.94E-01	3.05E-01	1.05	5.84E-01	7.72E-01	1.00
PC(15:0/22:6)	3.77E-01	5.26E-01	1.07	8.65E-01	9.34E-01	1.00
PC(16:0/12:0)	5.49E-01	6.80E-01	0.95	8.89E-01	9.41E-01	1.01
PC(16:0/14:0)	7.80E-01	8.39E-01	1.00	7.25E-01	8.63E-01	1.04
PC(16:0/15:0)	6.16E-02	1.15E-01	0.89	2.09E-01	4.10E-01	0.90
PC(16:0/16:0)	1.63E-02	3.71E-02	1.14	5.73E-02	1.35E-01	1.12
PC(16:0/16:1)	9.39E-02	1.68E-01	0.94	3.97E-01	6.15E-01	0.98
PC(16:0/17:0)	6.02E-01	7.11E-01	0.94	5.28E-01	7.31E-01	0.92
PC(16:0/17:1)	4.01E-01	5.50E-01	0.93	6.05E-01	7.89E-01	0.93
PC(16:0/18:1)	7.78E-02	1.42E-01	1.09	1.34E-01	2.78E-01	1.09
PC(16:0/18:2)	3.22E-02	6.85E-02	1.07	1.19E-01	2.53E-01	1.07
PC(16:0/18:3)	6.62E-01	7.52E-01	1.02	5.46E-01	7.46E-01	1.03
PC(16:0/19:1)	5.85E-01	7.08E-01	0.95	7.25E-01	8.63E-01	0.95
PC(16:0/20:3)	1.51E-02	3.48E-02	1.07	3.96E-02	9.95E-02	1.07
PC(16:0/20:4)	6.95E-05	3.06E-04	1.19	1.16E-02	3.43E-02	1.14
PC(16:0/20:5)	3.50E-01	5.01E-01	1.09	6.95E-01	8.40E-01	1.03
PC(16:0/22:1)	9.23E-01	9.52E-01	0.94	7.49E-01	8.80E-01	0.85
PC(16:0/22:4),PC(18:1/20:3)	5.09E-05	2.37E-04	1.21	4.98E-03	1.78E-02	1.16
PC(16:0_22:6)	5.17E-03	1.40E-02	1.05	1.17E-02	3.43E-02	1.06
PC(16:1/14:0)	7.61E-02	1.39E-01	0.90	3.98E-01	6.15E-01	0.95
PC(16:1/16:1)	9.31E-01	9.54E-01	1.02	3.95E-01	6.15E-01	1.07
PC(16:1/18:2)	8.06E-01	8.58E-01	1.00	6.16E-01	7.89E-01	1.03
PC(16:1/18:3)	3.20E-01	4.65E-01	1.06	2.62E-01	4.73E-01	1.09
PC(16:1/20:4)	5.73E-02	1.09E-01	0.95	9.34E-02	2.04E-01	0.96
PC(16:1/20:5)	1.55E-01	2.54E-01	0.95	6.34E-01	7.95E-01	0.98
PC(16:2/18:2)	6.51E-01	7.48E-01	1.01	5.10E-01	7.20E-01	1.01
PC(16:2/18:3)	9.66E-01	9.74E-01	1.01	3.82E-01	6.06E-01	1.07
PC(16:2/20:5)	2.88E-01	4.26E-01	1.06	2.74E-01	4.86E-01	1.07
PC(16:2/22:6)	3.54E-01	5.03E-01	1.06	8.67E-01	9.34E-01	1.02
PC(17:0/20:4)	2.69E-01	4.04E-01	1.06	7.61E-01	8.89E-01	1.00
PC(17:0/22:6)	5.97E-01	7.10E-01	1.02	9.97E-01	9.97E-01	1.00
PC(17:0_18:1)	2.91E-02	6.30E-02	0.89	5.11E-02	1.24E-01	0.87
PC(17:0_18:2),PC(15:0/20:2)	4.12E-01	5.61E-01	0.96	4.73E-01	6.86E-01	0.95

PC(17:1/18:2)	9.67E-01	9.74E-01	1.00	9.07E-01	9.53E-01	1.00
PC(18:0/16:0)	2.52E-04	1.01E-03	1.21	8.86E-03	2.86E-02	1.18
PC(18:0/18:0)	4.83E-03	1.33E-02	1.10	2.27E-02	6.02E-02	1.09
PC(18:0/18:1)	4.01E-02	8.22E-02	1.13	9.88E-02	2.12E-01	1.12
PC(18:0/18:2)	5.82E-02	1.10E-01	1.07	8.20E-02	1.81E-01	1.07
PC(18:0/19:1)	4.79E-01	6.18E-01	0.92	5.67E-01	7.56E-01	0.91
PC(18:0/20:1)	5.00E-01	6.36E-01	0.93	4.03E-01	6.15E-01	0.86
PC(18:0/20:3)	1.20E-01	2.05E-01	1.08	1.47E-01	3.00E-01	1.09
PC(18:0/20:4)	6.12E-05	2.77E-04	1.21	5.73E-03	2.00E-02	1.16
PC(18:0/20:5),PC(18:1/20:4)	2.33E-02	5.14E-02	1.14	2.41E-01	4.45E-01	1.07
PC(18:0/22:4)	2.37E-06	1.53E-05	1.25	1.18E-04	6.98E-04	1.24
PC(18:0/22:5)	9.09E-04	3.09E-03	1.16	1.86E-02	5.05E-02	1.13
PC(18:0/22:6)	1.47E-05	7.45E-05	1.31	8.22E-04	3.90E-03	1.22
PC(18:1/18:2)	4.47E-03	1.27E-02	1.14	2.27E-02	6.02E-02	1.14
PC(18:1/22:6)	4.51E-02	9.13E-02	1.06	2.27E-01	4.27E-01	1.05
PC(18:2/18:2)	5.73E-04	2.06E-03	1.18	2.79E-02	7.26E-02	1.12
PC(18:2_20:4)	8.47E-06	4.69E-05	1.29	2.06E-03	8.73E-03	1.21
PC(18:4/20:3)	2.80E-02	6.11E-02	1.05	8.07E-03	2.64E-02	1.08
PC(18:4/22:5)	5.61E-06	3.22E-05	1.12	1.31E-03	5.90E-03	1.10
PC(18:4/22:6)	9.45E-02	1.69E-01	1.11	3.50E-01	5.76E-01	1.06
PC(19:0/18:2),PC(17:0_20:2)	5.34E-02	1.05E-01	0.90	1.72E-01	3.44E-01	0.91
PC(19:0/20:4)	5.25E-01	6.58E-01	1.04	8.80E-01	9.41E-01	0.99
PC(19:1/18:2)	9.32E-01	9.54E-01	0.97	8.17E-01	9.20E-01	0.96
PC(19:1/22:6)	5.17E-01	6.50E-01	1.03	8.67E-01	9.34E-01	0.99
PC(20:0/18:2)	9.25E-03	2.26E-02	1.16	9.52E-02	2.07E-01	1.12
PC(20:0/20:4)	4.39E-06	2.60E-05	1.36	3.50E-04	1.84E-03	1.33
PC(20:0/22:4)	2.74E-05	1.32E-04	1.24	8.03E-05	5.18E-04	1.29
PC(20:0/22:5)	7.57E-01	8.24E-01	0.82	6.63E-01	8.16E-01	0.86
PC(20:0/22:6)	2.67E-03	8.24E-03	1.19	1.50E-02	4.23E-02	1.18
PC(20:1/18:2)	4.32E-03	1.24E-02	1.21	1.69E-02	4.67E-02	1.19
PC(20:1_20:4)	4.20E-04	1.56E-03	1.19	5.85E-03	2.01E-02	1.16
PC(20:3/20:4)	5.50E-02	1.06E-01	1.06	2.49E-01	4.58E-01	1.05
PC(20:4_22:6)	1.90E-03	6.08E-03	1.11	2.68E-02	7.01E-02	1.08
PC(20:5/18:2)	9.16E-03	2.25E-02	1.07	7.54E-03	2.49E-02	1.08
PC(20:5/22:6)	6.16E-05	2.77E-04	1.24	1.82E-03	7.87E-03	1.23
PC(24:0/20:4)	2.35E-08	2.54E-07	1.37	3.16E-06	3.59E-05	1.39
PC-O 32:0	4.53E-03	1.28E-02	1.17	1.49E-02	4.23E-02	1.16
PC-O 32:2	3.90E-02	8.03E-02	1.14	4.59E-02	1.13E-01	1.18
PC-O 34:1 (FA18:1)	1.68E-01	2.70E-01	1.09	3.14E-01	5.34E-01	1.07
PC-O 34:2 (FA18:2)	5.74E-03	1.54E-02	1.18	4.67E-03	1.72E-02	1.22
PC-O 34:3 (FA18:2)	2.25E-04	9.16E-04	1.24	8.22E-04	3.90E-03	1.29
PC-O 34:4	8.30E-04	2.91E-03	1.16	4.67E-03	1.72E-02	1.15
PC-O 35:3	5.55E-02	1.07E-01	1.12	6.29E-02	1.45E-01	1.13
PC-O 36:2 (FA18:2)	6.67E-03	1.75E-02	1.16	1.84E-02	5.02E-02	1.17
PC-O 36:3 (FA18:2)	8.57E-04	2.98E-03	1.21	1.38E-02	3.95E-02	1.22
PC-O 36:4 (FA20:3)	6.90E-07	5.06E-06	1.30	7.16E-05	4.96E-04	1.25
PC-O 36:5 (FA20:4)	6.82E-07	5.06E-06	1.29	3.19E-05	2.55E-04	1.28
PC-O 36:6 (FA20:4)	1.27E-03	4.16E-03	1.24	1.54E-02	4.33E-02	1.17
PC-O 38:3 (FA22:3)	9.53E-06	5.08E-05	1.27	5.47E-04	2.75E-03	1.26
PC-O 38:4 (FA20:4)	3.62E-08	3.72E-07	1.32	1.76E-05	1.61E-04	1.29
PC-O 38:5 (FA20:4)	1.43E-04	5.96E-04	1.20	3.50E-03	1.36E-02	1.15
PC-O 38:6 (FA22:6)	1.78E-05	8.96E-05	1.23	6.52E-04	3.20E-03	1.19
PC-O 38:7	9.30E-07	6.58E-06	1.16	7.65E-05	5.09E-04	1.15
PC-O 38:7 (FA22:6)	5.66E-06	3.22E-05	1.16	3.53E-04	1.84E-03	1.15
PC-O 40:4 (FA20:4)	5.27E-06	3.08E-05	1.20	6.14E-05	4.35E-04	1.20
PC-O 40:5 (FA22:5)	2.23E-03	6.92E-03	1.18	1.17E-02	3.43E-02	1.18
PC-O 40:6 (FA22:4)	5.07E-08	4.98E-07	1.31	2.74E-05	2.37E-04	1.27
PC-O 40:7 (FA22:5)	3.52E-08	3.71E-07	1.30	2.36E-05	2.08E-04	1.26
PC-O 40:8	5.39E-04	1.98E-03	1.10	7.29E-03	2.44E-02	1.09
PE(16:0/16:1)	3.36E-01	4.82E-01	1.04	6.73E-01	8.24E-01	1.03
PE(16:0/18:1)	9.67E-01	9.74E-01	1.02	9.83E-01	9.92E-01	0.98
PE(16:0/18:2)	5.48E-01	6.80E-01	1.05	6.60E-01	8.14E-01	1.03
PE(16:0/18:3)	1.61E-02	3.71E-02	0.89	6.93E-02	1.56E-01	0.87
PE(16:0/20:4)	4.52E-02	9.13E-02	1.13	3.26E-01	5.48E-01	1.06
PE(16:0/20:5)	5.38E-01	6.69E-01	1.00	3.14E-01	5.34E-01	0.88
PE(16:0/22:5)	4.87E-01	6.22E-01	0.97	1.23E-01	2.58E-01	0.88
PE(16:0/22:6)	1.77E-01	2.84E-01	1.23	7.63E-01	8.89E-01	1.01
PE(16:0p/18:2)	5.82E-03	1.55E-02	1.21	1.10E-02	3.33E-02	1.23
PE(16:0p/20:4)	1.09E-02	2.56E-02	1.12	2.99E-02	7.73E-02	1.08
PE(16:0p/20:5)	1.43E-01	2.36E-01	1.20	3.38E-01	5.61E-01	1.09
PE(16:0p/22:4)	2.32E-07	2.00E-06	1.36	7.88E-05	5.15E-04	1.28
PE(16:0p/22:5)	1.30E-01	2.19E-01	1.07	2.51E-01	4.60E-01	1.03
PE(16:0p/22:6)	7.33E-02	1.35E-01	1.12	2.21E-01	4.27E-01	1.06
PE(16:0p_18:1)	8.36E-02	1.51E-01	1.13	1.55E-01	3.14E-01	1.09
PE(16:1p/22:6)	3.64E-01	5.12E-01	1.00	8.02E-01	9.12E-01	0.99
PE(17:0/18:2)	3.08E-03	9.43E-03	0.82	4.32E-03	1.62E-02	0.79
PE(17:0/22:6)	2.04E-02	4.53E-02	0.88	3.33E-03	1.31E-02	0.78
PE(18:0/18:1)	7.78E-01	8.39E-01	0.99	9.75E-01	9.92E-01	0.92

PE(18:0/18:2)	1.89E-02	4.25E-02	1.16	9.56E-02	2.07E-01	1.11
PE(18:0/20:3)	4.26E-01	5.73E-01	1.05	9.01E-01	9.50E-01	0.99
PE(18:0/20:4)	3.22E-01	4.65E-01	1.06	9.79E-01	9.92E-01	1.00
PE(18:0/22:4)	1.96E-02	4.39E-02	1.14	3.51E-02	8.91E-02	1.12
PE(18:0/22:5),PE(18:1_22:4)	7.42E-01	8.16E-01	1.05	6.18E-01	7.89E-01	0.98
PE(18:0/22:6)	6.17E-01	7.18E-01	1.07	8.42E-01	9.30E-01	0.96
PE(18:0p/18:1)	1.12E-02	2.62E-02	1.16	6.76E-02	1.54E-01	1.13
PE(18:0p/20:4)	2.27E-07	2.00E-06	1.36	7.44E-05	5.02E-04	1.28
PE(18:0p/22:4)	1.46E-08	1.62E-07	1.39	1.12E-05	1.10E-04	1.35
PE(18:0p/22:5)	7.50E-03	1.91E-02	1.17	5.92E-02	1.39E-01	1.11
PE(18:0p/22:6)	8.86E-03	2.19E-02	1.16	5.19E-02	1.25E-01	1.12
PE(18:0p_18:2),PE(18:1p_18:1)	7.48E-04	2.65E-03	1.24	7.44E-03	2.47E-02	1.26
PE(18:1/18:2)	7.82E-01	8.39E-01	0.96	6.11E-01	7.89E-01	0.94
PE(18:1/20:3)	3.22E-01	4.65E-01	1.06	9.79E-01	9.92E-01	1.00
PE(18:1/20:4)	4.49E-01	5.92E-01	0.97	1.05E-01	2.24E-01	0.87
PE(18:1/22:4)	4.17E-01	5.66E-01	1.07	9.74E-01	9.92E-01	0.98
PE(18:1/22:6)	3.81E-02	7.92E-02	1.18	6.10E-01	7.89E-01	1.05
PE(18:1p/18:2)	7.80E-03	1.96E-02	1.21	3.65E-02	9.21E-02	1.18
PE(18:1p/20:3)	8.78E-04	3.01E-03	1.17	1.01E-02	3.14E-02	1.12
PE(18:1p/20:4)	1.76E-03	5.67E-03	1.21	1.62E-02	4.53E-02	1.14
PE(18:1p/22:4)	7.39E-03	1.89E-02	1.17	5.98E-02	1.40E-01	1.12
PE(18:1p/22:6)	7.52E-01	8.21E-01	1.04	8.60E-01	9.34E-01	0.98
PE(18:1p_20:5),PE(18:2p_20:4)	4.14E-06	2.52E-05	1.34	5.80E-04	2.88E-03	1.25
PE(18:2/18:2)	8.85E-02	1.59E-01	1.12	3.36E-01	5.61E-01	1.06
PE(18:2/20:4)	1.80E-01	2.87E-01	1.23	7.72E-01	8.93E-01	1.01
PE(18:2p/18:2)	5.18E-01	6.50E-01	1.05	3.98E-01	6.15E-01	1.03
PE(18:2p/22:4)	9.52E-03	2.29E-02	1.16	5.30E-02	1.27E-01	1.12
PE(18:2p/22:6)	4.72E-01	6.18E-01	1.06	9.30E-01	9.66E-01	0.99
PE(20:0p/18:2)	1.10E-05	5.74E-05	1.31	3.09E-05	2.55E-04	1.39
PE(20:0p/20:4)	8.07E-09	9.18E-08	1.40	8.02E-06	8.45E-05	1.36
PE(20:0p/22:6)	1.07E-02	2.53E-02	1.24	5.33E-02	1.27E-01	1.15
PE(20:0p_20:5), PE(18:0p/22:5)	1.77E-07	1.62E-06	1.42	1.87E-04	1.09E-03	1.32
PE(20:1p/20:4)	1.26E-01	2.13E-01	1.12	2.15E-01	4.16E-01	1.06
PE(20:1p/22:6)	9.86E-01	9.88E-01	1.05	4.90E-01	7.05E-01	0.95
PG(18:0/18:1)	3.80E-02	7.92E-02	1.14	2.23E-01	4.27E-01	1.08
PI(16:0/18:2)	4.36E-03	1.25E-02	1.15	4.84E-03	1.75E-02	1.16
PI(16:0/20:3)	2.33E-01	3.58E-01	1.06	5.22E-01	7.27E-01	1.01
PI(16:0/22:6)	3.64E-03	1.07E-02	1.16	3.37E-02	8.61E-02	1.11
PI(17:0/18:2)	4.34E-01	5.79E-01	0.88	6.43E-01	8.01E-01	0.85
PI(17:0/20:4)	4.78E-01	6.18E-01	0.90	5.49E-01	7.48E-01	0.88
PI(18:0/18:1)	8.10E-01	8.60E-01	0.99	9.30E-01	9.66E-01	0.98
PI(18:0/20:3)	5.68E-01	6.92E-01	0.91	7.12E-01	8.52E-01	0.92
PI(18:0/22:4)	1.25E-01	2.12E-01	1.06	2.56E-01	4.66E-01	1.04
PI(18:1/20:4)	1.07E-01	1.85E-01	1.05	1.23E-01	2.58E-01	1.04
PI(18:1_18:1)	1.17E-01	2.01E-01	1.10	2.57E-01	4.66E-01	1.08
PI(18:2/18:2)	8.62E-04	2.98E-03	1.18	3.99E-03	1.54E-02	1.17
PS(20:0/18:2)	9.54E-03	2.29E-02	0.91	2.41E-01	4.45E-01	0.94
PS(21:0/22:5)	1.63E-02	3.71E-02	1.09	1.46E-01	3.00E-01	1.05
SM 32:0	8.76E-06	4.79E-05	1.25	9.42E-05	5.99E-04	1.26
SM 33:1	2.90E-07	2.36E-06	1.30	3.70E-05	2.90E-04	1.27
SM 34:0	1.15E-11	1.78E-10	1.28	1.61E-08	2.39E-07	1.28
SM 36:0	9.73E-03	2.32E-02	1.10	1.59E-01	3.20E-01	1.04
SM 36:3	4.42E-09	5.31E-08	1.15	2.00E-07	2.70E-06	1.15
SM 36:4	3.86E-08	3.88E-07	1.36	6.08E-06	6.56E-05	1.35
SM 38:3	9.79E-11	1.36E-09	1.41	1.04E-07	1.45E-06	1.38
SM 38:5	4.11E-06	2.52E-05	1.43	5.30E-03	1.88E-02	1.29
SM 40:1	1.78E-15	4.26E-14	1.37	7.10E-12	1.62E-10	1.39
SM 40:2	7.27E-13	1.26E-11	1.37	2.33E-09	3.73E-08	1.38
SM 42:4	3.04E-07	2.43E-06	1.32	2.08E-04	1.16E-03	1.30
SM(d16:1/16:0)	2.68E-06	1.68E-05	1.27	5.11E-05	3.74E-04	1.28
SM(d16:1/16:1)	7.23E-03	1.86E-02	1.12	1.91E-02	5.11E-02	1.14
SM(d18:1/16:0)	3.93E-12	6.30E-11	1.34	5.31E-08	7.65E-07	1.32
SM(d18:1/18:0)	5.02E-10	6.58E-09	1.32	1.38E-06	1.65E-05	1.27
SM(d18:1/20:0)	1.00E-04	4.29E-04	1.25	2.36E-03	9.81E-03	1.19
SM(d18:1/24:0)	3.33E-15	7.57E-14	1.39	5.31E-11	1.15E-09	1.41
SM(d18:2/16:0)	1.62E-06	1.09E-05	1.30	2.10E-04	1.16E-03	1.25
SM(d18:2/18:0)	1.88E-10	2.53E-09	1.28	9.91E-06	9.96E-05	1.23
SM(d18:2/20:0)	6.88E-07	5.06E-06	1.26	1.14E-04	6.82E-04	1.23
SM(d18:2/24:1)	8.76E-01	9.16E-01	1.03	9.09E-01	9.53E-01	0.99
SM(d22:1/18:0)	2.15E-06	1.40E-05	1.21	5.06E-06	5.60E-05	1.26
So(d18:0)	2.13E-01	3.30E-01	0.95	3.50E-01	5.76E-01	0.99
TG(10:0/16:0/18:1)	9.43E-01	9.62E-01	1.01	7.96E-01	9.12E-01	0.90
TG(10:0/16:0/18:2)	9.57E-01	9.70E-01	1.02	5.07E-01	7.20E-01	0.82
TG(10:0/16:1/18:2)	9.29E-01	9.54E-01	1.15	4.04E-01	6.15E-01	0.75
TG(10:0/18:1/18:2)	7.81E-01	8.39E-01	0.91	4.94E-01	7.07E-01	0.73
TG(10:0/18:2/18:2)	4.62E-03	1.29E-02	0.89	4.90E-02	1.19E-01	0.91
TG(12:0/18:2/20:4)	8.00E-01	8.53E-01	0.96	9.21E-01	9.61E-01	0.71
TG(12:0/18:2/22:6)	8.14E-01	8.62E-01	0.94	6.21E-01	7.91E-01	0.87

TG(13:0/18:2/18:2)	7.53E-01	8.21E-01	1.07	9.97E-01	9.97E-01	0.86
TG(14:0/18:2/18:3)	7.17E-01	7.99E-01	1.03	8.87E-01	9.41E-01	0.79
TG(14:0/18:2/20:4)	6.08E-01	7.13E-01	1.00	8.05E-01	9.12E-01	0.89
TG(14:0/18:2/22:6),TG(16:0/16:2/22:6)	2.58E-01	3.92E-01	1.13	4.56E-01	6.73E-01	0.93
TG(14:0/18:3/18:3)	6.37E-02	1.18E-01	0.89	1.42E-01	2.92E-01	0.88
TG(15:0/14:0/18:2)	8.52E-01	8.98E-01	0.97	9.88E-01	9.95E-01	0.90
TG(15:0/18:1/18:2)	7.35E-01	8.12E-01	0.92	5.44E-01	7.46E-01	0.83
TG(15:0/18:1/20:4)	5.88E-01	7.08E-01	0.97	8.58E-01	9.34E-01	0.87
TG(15:0/18:2/18:2)	4.77E-01	6.18E-01	0.98	7.48E-01	8.80E-01	0.87
TG(15:0/18:2/18:3)	1.37E-01	2.29E-01	1.14	4.73E-01	6.86E-01	0.96
TG(15:0/18:3/20:4),TG(15:0/18:2/20:5)	9.04E-01	9.36E-01	0.99	8.24E-01	9.20E-01	0.98
TG(16:0/12:0/14:0)	8.84E-01	9.21E-01	1.01	6.80E-01	8.27E-01	0.90
TG(16:0/12:0/20:4)	7.53E-01	8.21E-01	1.00	6.98E-01	8.40E-01	0.78
TG(16:0/14:0/14:0)	9.44E-01	9.62E-01	1.05	6.00E-01	7.88E-01	0.97
TG(16:0/14:0/16:0)	5.73E-01	6.96E-01	0.99	9.81E-01	9.92E-01	0.87
TG(16:0/14:0/16:1)	8.69E-01	9.12E-01	1.01	7.98E-01	9.12E-01	0.93
TG(16:0/14:0/18:1)	6.59E-01	7.52E-01	0.95	9.63E-01	9.88E-01	0.88
TG(16:0/14:0/18:3),TG(16:1/14:0/18:2)	5.98E-01	7.10E-01	1.02	5.72E-01	7.58E-01	0.90
TG(16:0/14:0/20:4),TG(14:0/18:2/18:2)	1.89E-01	2.99E-01	1.03	3.24E-01	5.47E-01	0.93
TG(16:0/14:0/20:5)	6.94E-01	7.81E-01	1.04	8.89E-01	9.41E-01	0.79
TG(16:0/14:0/22:6)	6.89E-01	7.77E-01	0.99	8.69E-01	9.34E-01	0.89
TG(16:0/15:0/16:0)	5.59E-01	6.90E-01	1.00	5.58E-01	7.48E-01	0.96
TG(16:0/15:0/16:1),TG(15:0/14:0/18:1)	2.98E-01	4.37E-01	0.97	5.20E-01	7.27E-01	0.94
TG(16:0/15:0/18:1)	4.43E-01	5.85E-01	0.95	5.55E-01	7.48E-01	0.86
TG(16:0/15:0/18:2)	5.67E-01	6.92E-01	0.95	6.29E-01	7.94E-01	0.87
TG(16:0/15:0/22:6)	4.37E-01	5.81E-01	1.05	6.50E-01	8.05E-01	0.92
TG(16:0/15:1/18:2)	5.87E-01	7.08E-01	0.92	7.61E-01	8.89E-01	0.85
TG(16:0/16:0/16:0)	7.06E-01	7.90E-01	1.09	6.26E-01	7.93E-01	0.90
TG(16:0/16:0/17:0)	5.34E-01	6.67E-01	0.98	8.32E-01	9.23E-01	0.88
TG(16:0/16:0/19:0)	5.00E-01	6.36E-01	0.90	5.60E-01	7.48E-01	0.80
TG(16:0/16:0/20:3),TG(16:0/18:1/18:2)	9.88E-02	1.74E-01	1.04	3.74E-01	6.01E-01	0.96
TG(16:0/16:1/16:1),TG(16:0/14:0/18:2)	9.78E-01	9.82E-01	0.98	7.08E-01	8.50E-01	0.90
TG(16:0/16:1/18:1)	7.06E-01	7.90E-01	1.01	7.72E-01	8.93E-01	0.91
TG(16:0/16:1/18:2)	6.38E-01	7.41E-01	1.00	6.68E-01	8.20E-01	0.92
TG(16:0/17:0/18:1)	5.69E-01	6.92E-01	0.88	5.26E-01	7.31E-01	0.77
TG(16:0/17:0/18:2)	7.31E-01	8.09E-01	0.91	5.09E-01	7.20E-01	0.81
TG(16:0/17:1/19:1)	1.58E-01	2.58E-01	1.02	3.99E-01	6.15E-01	0.94
TG(16:0/18:1/18:1)	4.41E-01	5.84E-01	0.98	7.87E-01	9.05E-01	0.90
TG(16:0/18:1/20:4)	5.45E-02	1.06E-01	1.03	2.69E-01	4.79E-01	0.93
TG(16:0/18:1/22:0)	6.56E-01	7.52E-01	0.79	3.66E-01	5.94E-01	0.68
TG(16:0/18:1/22:1)	7.26E-01	8.07E-01	0.63	9.00E-01	9.50E-01	0.55
TG(16:0/18:1/22:4)	1.23E-01	2.10E-01	1.03	4.11E-01	6.23E-01	0.93
TG(16:0/18:1/22:5)	1.96E-01	3.07E-01	1.02	6.13E-01	7.89E-01	0.91
TG(16:0/18:1/22:6)	9.94E-02	1.75E-01	1.04	5.50E-01	7.48E-01	0.90
TG(16:0/18:2/18:2),TG(16:1/18:1/18:2)	3.86E-02	7.97E-02	1.08	2.13E-01	4.16E-01	0.99
TG(16:0/18:2/18:3)	3.88E-01	5.37E-01	0.99	5.70E-01	7.58E-01	0.90
TG(16:0/18:2/20:4)	1.51E-01	2.48E-01	1.01	3.94E-01	6.15E-01	0.91
TG(16:0/18:2/20:5),TG(18:3/18:2/18:2)	3.52E-01	5.02E-01	1.00	4.45E-01	6.61E-01	0.91
TG(16:0/18:2/22:5)	1.04E-01	1.81E-01	1.04	5.60E-01	7.48E-01	0.90
TG(16:0/18:2/22:6)	2.99E-02	6.40E-02	1.17	1.85E-01	3.65E-01	0.99
TG(16:0/18:3/22:6)	3.92E-01	5.40E-01	1.13	8.44E-01	9.30E-01	0.94
TG(16:0/20:4/22:6)	5.78E-02	1.10E-01	1.25	4.28E-01	6.46E-01	1.03
TG(16:0/22:4/22:6)	4.34E-02	8.85E-02	1.16	3.88E-01	6.14E-01	1.04
TG(16:0/22:5/22:5)	3.42E-02	7.17E-02	1.16	3.22E-01	5.45E-01	1.05
TG(16:0/22:6/22:6)	5.28E-02	1.05E-01	1.28	3.10E-01	5.34E-01	1.06
TG(16:1/12:0/18:1),TG(16:0/12:0/18:2)	6.47E-01	7.48E-01	1.04	5.17E-01	7.27E-01	0.89
TG(16:1/12:0/18:2)	7.81E-01	8.39E-01	0.91	4.94E-01	7.07E-01	0.73
TG(16:1/14:0/18:3)	8.58E-01	9.02E-01	0.98	7.87E-01	9.05E-01	0.76
TG(16:1/16:1/18:1)	3.56E-01	5.04E-01	1.03	4.02E-01	6.15E-01	0.95
TG(16:1/18:2/18:3)	6.89E-01	7.77E-01	0.99	8.69E-01	9.34E-01	0.89
TG(16:1/18:2/20:4)	3.63E-01	5.12E-01	1.01	4.51E-01	6.67E-01	0.91
TG(16:1/18:2/20:5)	6.11E-01	7.15E-01	1.07	6.18E-01	7.89E-01	0.89
TG(16:1/18:2/22:5)	3.24E-02	6.87E-02	1.16	1.84E-01	3.64E-01	0.99
TG(14:0/20:4/22:6),TG(18:3/18:2/20:5)	6.02E-01	7.11E-01	1.08	4.44E-01	6.61E-01	0.90
TG(16:2/18:2/18:3)	5.95E-01	7.10E-01	1.03	8.16E-01	9.20E-01	0.85
TG(17:0/18:1/18:1)	5.17E-01	6.50E-01	0.81	2.96E-01	5.15E-01	0.70
TG(17:0/18:1/18:2)	9.55E-01	9.70E-01	0.89	6.39E-01	7.97E-01	0.79
TG(17:0/18:1/20:4)	9.93E-01	9.93E-01	0.96	5.34E-01	7.34E-01	0.89
TG(17:0/18:2/20:4)	6.06E-01	7.13E-01	1.04	8.76E-01	9.39E-01	0.92
TG(17:0/18:3/18:3)	8.94E-01	9.28E-01	0.99	8.03E-01	9.12E-01	0.99
TG(17:1/18:1/18:2)	8.42E-01	8.89E-01	0.90	6.30E-01	7.94E-01	0.81
TG(18:0/16:0/16:0)	4.34E-01	5.79E-01	1.13	4.66E-01	6.82E-01	0.88
TG(18:0/16:0/18:1)	2.41E-01	3.69E-01	1.02	3.57E-01	5.85E-01	0.87
TG(18:0/17:0/18:1)	5.96E-01	7.10E-01	0.85	2.81E-01	4.96E-01	0.73
TG(18:0/18:0/18:1)	3.79E-01	5.26E-01	0.94	6.50E-01	8.05E-01	0.84
TG(18:0/18:0/20:4)	4.75E-01	6.18E-01	0.92	8.26E-01	9.20E-01	0.83
TG(18:0/18:1/18:1),TG(16:0/18:1/20:1)	2.76E-01	4.13E-01	0.90	6.22E-01	7.91E-01	0.78
TG(18:0/18:1/20:3)	4.83E-01	6.20E-01	0.92	8.23E-01	9.20E-01	0.83

TG(18:0/18:1/20:4)	1.20E-01	2.05E-01	1.03	4.03E-01	6.15E-01	0.93
TG(18:0/18:1/22:4)	6.81E-01	7.72E-01	0.77	7.35E-01	8.68E-01	0.68
TG(18:0/18:1/22:5)	5.65E-01	6.92E-01	0.94	9.56E-01	9.83E-01	0.84
TG(18:0/18:1/22:6)	3.79E-01	5.26E-01	1.01	8.55E-01	9.34E-01	0.88
TG(18:0/18:2/20:3)	7.93E-02	1.44E-01	1.04	3.72E-01	6.01E-01	0.93
TG(18:0/18:2/20:4)	1.98E-01	3.09E-01	1.02	6.14E-01	7.89E-01	0.91
TG(18:0/20:4/20:4)	5.38E-02	1.05E-01	1.10	2.41E-01	4.45E-01	0.97
TG(18:0/20:4/22:6)	2.93E-02	6.30E-02	1.16	2.96E-01	5.15E-01	1.05
TG(18:0/22:6/22:6)	4.96E-02	9.92E-02	1.18	3.42E-01	5.66E-01	1.03
TG(18:1/18:1/18:2)	5.74E-02	1.09E-01	1.01	3.12E-01	5.34E-01	0.91
TG(18:1/18:1/20:4)	5.64E-01	6.92E-01	0.98	9.52E-01	9.83E-01	0.86
TG(18:1/18:1/22:1)	2.63E-01	3.98E-01	0.55	5.09E-01	7.20E-01	0.57
TG(18:1/18:1/22:2)	1.46E-01	2.40E-01	0.56	2.27E-01	4.27E-01	0.54
TG(18:1/18:1/22:3)	5.07E-01	6.42E-01	0.78	4.44E-01	6.61E-01	0.69
TG(18:1/18:1/22:5)	3.74E-01	5.24E-01	1.01	8.24E-01	9.20E-01	0.88
TG(18:1/18:1/22:6)	5.38E-02	1.05E-01	1.10	2.41E-01	4.45E-01	0.97
TG(18:1/18:1/24:0)	4.28E-01	5.74E-01	0.90	2.14E-01	4.16E-01	0.91
TG(18:1/18:2/18:2)	5.49E-02	1.06E-01	1.03	2.64E-01	4.76E-01	0.93
TG(18:1/18:2/19:0)	5.93E-01	7.10E-01	0.82	3.97E-01	6.15E-01	0.69
TG(18:1/18:2/20:2)	2.99E-01	4.37E-01	1.00	9.52E-01	9.83E-01	0.88
TG(18:1/18:2/20:3)	2.81E-01	4.18E-01	1.04	6.03E-01	7.89E-01	0.93
TG(18:1/18:2/20:4)	1.04E-01	1.81E-01	1.04	5.60E-01	7.48E-01	0.90
TG(18:1/18:2/22:0)	2.94E-01	4.34E-01	0.61	6.96E-01	8.40E-01	0.56
TG(18:1/18:2/22:1)	3.09E-01	4.52E-01	0.63	4.45E-01	6.61E-01	0.56
TG(18:1/18:2/22:4)	4.56E-01	5.99E-01	0.98	8.60E-01	9.34E-01	0.89
TG(18:1/18:2/22:5)	9.55E-02	1.69E-01	1.07	4.73E-01	6.86E-01	0.98
TG(18:1/20:4/20:4),TG(18:1/18:2/22:6)	6.22E-03	1.64E-02	1.19	8.99E-02	1.97E-01	1.04
TG(18:1/20:4/22:4),TG(18:1/20:2/22:6)	2.86E-01	4.25E-01	1.06	6.35E-01	7.95E-01	0.94
TG(18:1/20:4/22:5)	1.59E-01	2.58E-01	1.16	6.88E-01	8.35E-01	1.07
TG(18:1/20:4/22:6)	2.87E-02	6.24E-02	1.23	2.25E-01	4.27E-01	1.11
TG(18:1/22:6/22:6)	6.97E-02	1.29E-01	1.42	5.31E-01	7.33E-01	1.25
TG(18:2/18:2/18:2)	1.36E-01	2.29E-01	1.01	3.80E-01	6.06E-01	0.91
TG(18:2/18:2/22:6)	5.97E-02	1.12E-01	1.25	4.36E-01	6.56E-01	1.03
TG(18:2/20:4/22:6)	5.89E-02	1.11E-01	1.28	3.34E-01	5.60E-01	1.06
TG(18:2/22:6/22:6)	5.37E-02	1.05E-01	1.73	3.61E-01	5.89E-01	1.57
TG(18:3/18:2/20:4)	4.24E-01	5.72E-01	1.13	8.48E-01	9.32E-01	0.94
TG(20:0/18:1/18:2)	7.90E-01	8.45E-01	0.74	7.32E-01	8.68E-01	0.65
TG(20:0/18:1/20:4)	8.79E-01	9.17E-01	0.74	9.90E-01	9.95E-01	0.66
TG(20:1/18:1/18:1)	6.49E-01	7.48E-01	0.73	6.76E-01	8.25E-01	0.67
TG(20:1/18:1/18:2)	4.73E-01	6.18E-01	0.91	8.07E-01	9.12E-01	0.83
TG(20:1/18:1/20:4)	6.16E-01	7.18E-01	0.95	9.74E-01	9.92E-01	0.87
TG(20:3/18:2/18:2)	4.64E-02	9.32E-02	1.04	3.12E-01	5.34E-01	0.90
TG(20:3/18:2/20:3)	2.76E-02	6.04E-02	1.16	1.27E-01	2.65E-01	1.05
TG(20:5/18:2/18:2)	4.23E-01	5.72E-01	1.13	8.42E-01	9.30E-01	0.94
TG(20:5/18:2/20:4),TG(18:3/18:2/22:6)	6.59E-01	7.52E-01	1.32	8.87E-01	9.41E-01	1.12
TG(22:5/18:2/18:2)	3.49E-03	1.04E-02	1.20	6.39E-02	1.47E-01	1.05
TG(22:5/18:2/20:4)	3.26E-02	6.88E-02	1.23	2.65E-01	4.76E-01	1.10
